# Impaired axonal transport contributes to neurodegeneration in a Cre-inducible mouse model of myocilin-associated glaucoma

**DOI:** 10.1172/jci.insight.188710

**Published:** 2025-01-21

**Authors:** Balasankara Reddy Kaipa, Ramesh Kasetti, Yogapriya Sundaresan, Linya Li, Sam Yacoub, J. Cameron Millar, William Cho, Dorota Skowronska-Krawczyk, Prabhavathi Maddineni, Krzysztof Palczewski, Gulab S. Zode

**Affiliations:** 1 Gavin Herbert Eye Institute-Center for Translational Vision Research, Department of Ophthalmology, and; 2 Department of Physiology and Biophysics, University of California Irvine School of Medicine, Irvine, California, USA.; 3 Department of Pharmacology and Neuroscience, North Texas Eye Research Institute, University of North Texas Health Science Center at Fort Worth, Texas, USA.; 4 Department of Ophthalmology, School of Medicine, University of Missouri, Columbia, Missouri, USA.; 5 Department of Chemistry and; 6 Department of Molecular Biology and Biochemistry, University of California, Irvine, Irvine, California, USA.

**Keywords:** Genetics, Neuroscience, Ophthalmology, Neurodegeneration, Protein misfolding, Transport

## Abstract

Elevation of intraocular pressure (IOP) due to trabecular meshwork (TM) dysfunction, leading to neurodegeneration, is the pathological hallmark of primary open-angle glaucoma (POAG). Impaired axonal transport is an early and critical feature of glaucomatous neurodegeneration. However, a robust mouse model that accurately replicates these human POAG features has been lacking. We report the development and characterization of a new Cre-inducible mouse model expressing a DsRed-tagged Y437H mutant of human myocilin (*Tg.CreMYOC^Y437H^*). A single intravitreal injection of HAd5-Cre induced selective MYOC expression in the TM, causing TM dysfunction, reducing the outflow facility, and progressively elevating IOP in *Tg.CreMYOC^Y437H^* mice. Sustained IOP elevation resulted in significant loss of retinal ganglion cells (RGCs) and progressive axonal degeneration in Cre-induced *Tg.CreMYOC^Y437H^* mice. Notably, impaired anterograde axonal transport was observed at the optic nerve head before RGC degeneration, independent of age, indicating that impaired axonal transport contributes to RGC degeneration in *Tg.CreMYOC^Y437H^* mice. In contrast, axonal transport remained intact in ocular hypertensive mice injected with microbeads, despite significant RGC loss. Our findings indicate that Cre-inducible *Tg.CreMYOC^Y437H^* mice replicate all glaucoma phenotypes, providing an ideal model for studying early events of TM dysfunction and neuronal loss in POAG.

## Abstract 2

In this study, we developed and characterized a new mouse model that accurately replicates human glaucoma. Using this model, we further showed that sustained ocular hypertension impairs axonal transport at the optic nerve head prior to neuronal loss. This model provides a novel tool for studying glaucoma pathology and for identifying targets for developing new therapies.

## Graphical abstract

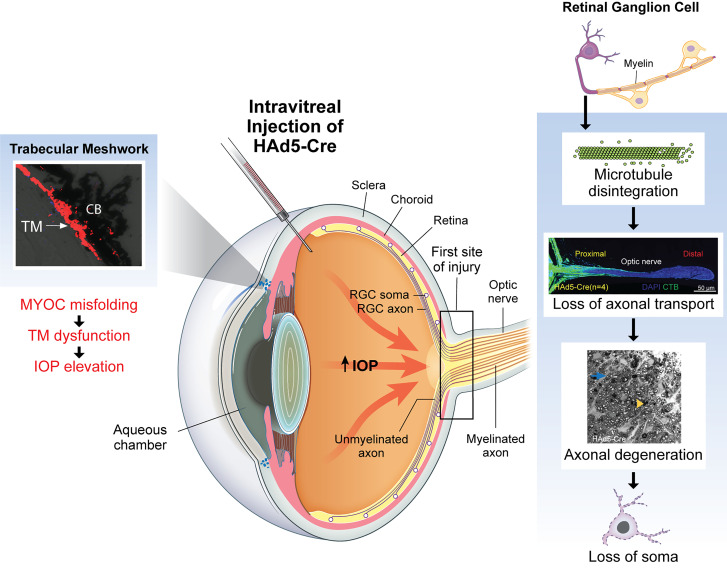

## Introduction

Glaucoma is a group of multifactorial neurodegenerative diseases characterized by progressive optic neuropathy. It is the second leading cause of irreversible vision loss, affecting more than 70 million people worldwide (1, 2), and this number is estimated to increase to 112 million by the year 2040 (3). Primary open-angle glaucoma (POAG) is the most common form of glaucoma, accounting for approximately 70% of all cases (1). POAG is characterized by progressive loss of the soma and axons of retinal ganglion cells (RGCs), leading to irreversible vision loss (1, 2). Impaired axonal transport at the optic nerve head (ONH) is implicated as an early pathological event associated with glaucomatous neurodegeneration (4–6). Elevated intraocular pressure (IOP) is a significant risk factor for POAG and the only one that is treatable (7). The trabecular meshwork (TM), a molecular sieve-like structure, regulates IOP by constantly adjusting the resistance to aqueous humor (AH) outflow. In POAG, increased resistance to AH outflow elevates IOP, leading to neurodegeneration (8–10). This increase in outflow resistance is associated with TM dysfunction (11–15). Most current treatment strategies for POAG do not target the underlying pathology of the TM and RGCs, and vision loss continues to progress in some patients (16). To understand the pathological mechanisms of TM dysfunction/IOP elevation and glaucomatous neurodegeneration, there is an unmet need to develop a simple and reliable animal model that can faithfully replicate all features of POAG.

Currently, several mouse models of ocular hypertension (OHT) are utilized to study the pathophysiology of glaucomatous neurodegeneration (17, 18). These include DBA/2J mice and inducible mouse models that physically block AH outflow through the TM, leading to OHT and neuronal loss (19–24). While these models have provided important mechanistic insights, they do not allow the study of TM pathology, as they elevate IOP by physically blocking the outflow pathway (21–23). Thus, these models do not truly represent the human-POAG phenotype, where the AH outflow pathway is open. Importantly, these models destroy the TM, which can induce acute and sudden IOP-induced glaucomatous neurodegeneration. Moreover, these models are often technically challenging to replicate in laboratory settings, and they exhibit variable phenotypes and other confounding features such as ocular inflammation. Developing a mouse model that mimics a known genetic cause of human POAG represents an ideal strategy for understanding the pathophysiology of POAG.

Mutation of myocilin (*MYOC*) is the most common genetic cause of POAG and significantly contributes to juvenile-onset OAG (JOAG) (9, 25, 26). MYOC-associated JOAG is a more aggressive form of glaucoma, characterized by high IOP in young children and rapid progression to vision loss (9, 26). Mouse models with these genetic alterations in the *MYOC* gene mimicking human POAG have proved to be invaluable tools for understanding the pathogenesis of POAG and designing treatment strategies (27–29). Previously, we developed a transgenic mouse model (*Tg-MYOC^Y437H^*) of myocilin POAG by random genomic insertion of the human mutant myocilin and demonstrated that *Tg-MYOC^Y437H^* mice develop glaucoma phenotypes closely resembling those seen in patients with POAG with the Y437H *MYOC* mutation (29). However, *Tg-MYOC^Y437H^* mice presented a few drawbacks, including (a) possible unknown gene mutations and multiple copies of the transgene due to random integration; (b) a mild phenotype on a pure C57BL/6J background, limiting the study of glaucomatous neurodegeneration; (c) possible silencing of the transgene upon subsequent breeding; and (d) lack of specific antibodies to detect myocilin in the mouse TM. To overcome these limitations, we utilized a TARGATT site-specific knock-in strategy (30) to generate transgenic mice expressing human mutant *MYOC*. This technology employs serine integrase, PhiC31 (ΦC31), to insert a single copy of a gene of interest into a preselected intergenic and transcriptionally active genomic locus (H11), which has been engineered with a docking site. This allows stable and single site-specific transgene integration. Since mutant MYOC is toxic to TM cells (29, 31), we exploited the Cre-lox system to develop an inducible mouse model in which mutant MYOC is expressed in the tissue of interest only upon Cre expression. Here, we report the development and characterization of a Cre-inducible transgenic mouse line expressing the DsRed-tagged Y437H mutant of human myocilin (*Tg.CreMYOC^Y437H^*). We further utilized this model to investigate early events of glaucomatous TM dysfunction and neurodegeneration. In contrast to the microbead-occlusion (MB-occlusion) model of OHT, in which axonal transport remains intact despite significant loss of RGCs, we observed that sustained IOP elevation in the *Tg.CreMYOC^Y437H^* mice significantly impairs axonal transport at the ONH prior to any loss of RGC somas and axons.

## Results

### Generation of an inducible mouse model of myocilin POAG.

**Figure 1:**
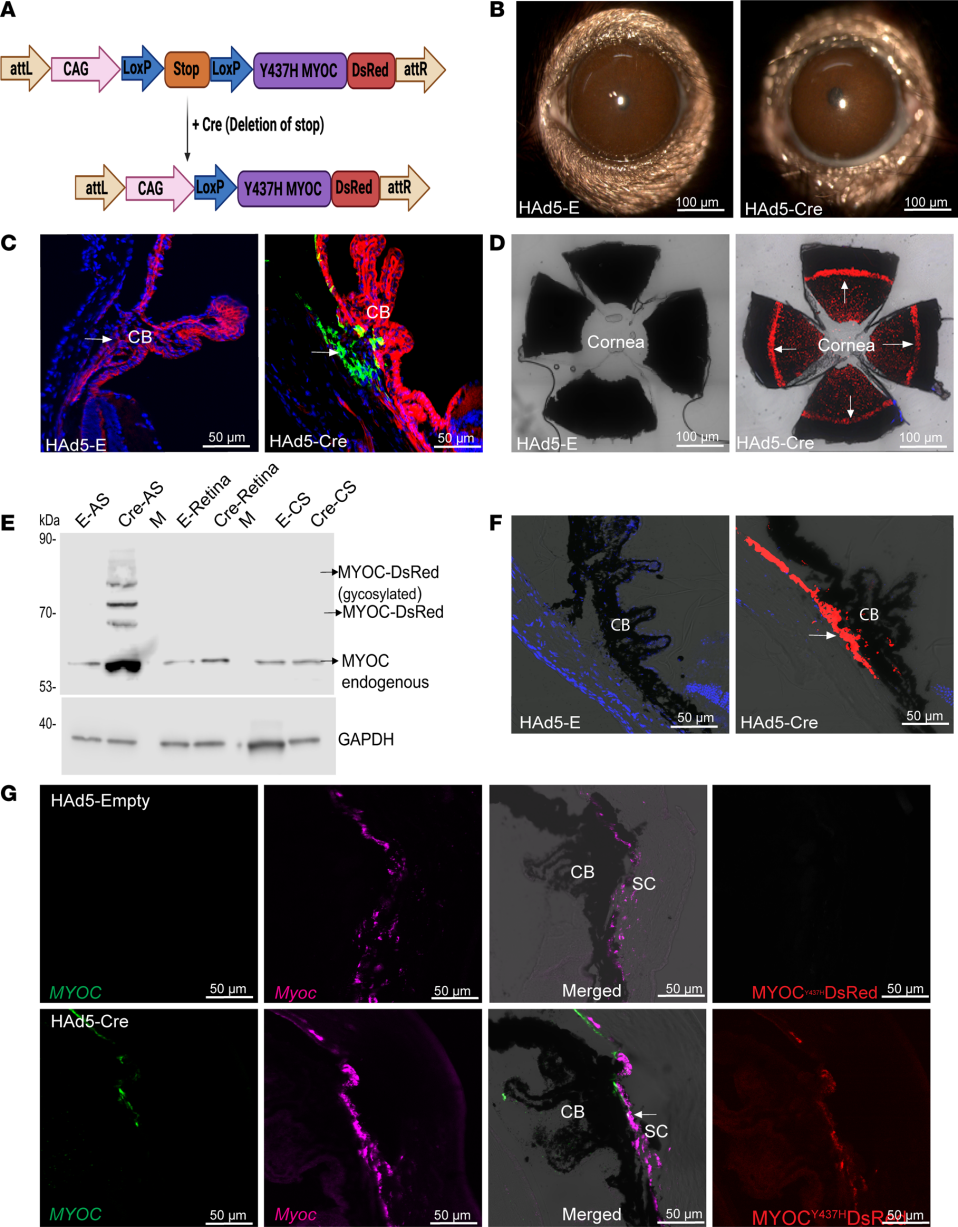
HAd5-Cre recombinase induces mutant MYOC in mouse TM. (**A**) *Tg.CreMYOC^Y437H^* mice were engineered using a TARGATT gene knock-in strategy in which DsRed-tagged human Y437H-mutant *MYOC* was inserted in a transcriptionally active genomic locus (H11). A stop cassette prevents the expression of the mutant MYOC-DsRed fusion protein until Cre recombinase is introduced. Following Cre expression, the stop cassette is excised, allowing the expression of the mutant *MYOC* fused with DsRed only in targeted cells. (**B**) Representative slit-lamp images showing that no obvious ocular inflammation is associated with either HAd5-Empty– or HAd5-Cre–injected *Tg.CreMYOC^Y437H^* mice (*n* = 6). Scale bars: 100 μm. (**C**) HAd5-Cre was injected intravitreally (2 × 10^7^ pfu/eye) in mT/mG fluorescence-based reporter mice, and the conversion from tdTomato to GFP was examined 1 week after injection using confocal microscopy (*n* = 4). (**D**–**F**) *Tg.CreMYOC^Y437H^* mice were injected intravitreally with HAd5-Empty or HAd5-Cre, and MYOC induction was examined in the TM using confocal imaging of DsRed protein in whole mount anterior segment (scale bars: 100 μm) (**D**), Western blot analysis of various ocular tissues with MYOC-antibody (**E**) showing the presence of human mutant myocilin in anterior-segment tissues of Cre-injected eyes (8 weeks after injection; AS, anterior segment; M, empty lane; CS, choroid and sclera); and confocal imaging of DsRed protein in the anterior-segment cross-section from Cre^–^- and Cre^+^-*Tg.CreMYOC^Y437H^* mice (**F**) (*n* = 4). Scale bars: 50 μm.. (**G**) RNAScope analysis of *MYOC* (green) and *Myoc* (pink) transcripts in the anterior-segment cross-section of Cre^–^- and Cre^+^-*Tg.CreMYOC^Y437H^* mice. The last panel shows DsRed-protein expression on the same slide. Note that DsRed fluorescence may appear to be less intense compared with other images, due to fluorescence quenching during sample processing (TM, trabecular meshwork; CB, ciliary body; SC, Schlemm’s canal). Arrows show the TM. Scale bars: 50 μm.

Using the TARGATT site-specific knock-in strategy, we developed Cre-inducible transgenic mice that express the DsRed-tagged Y437H-mutant of human *MYOC* (referred to as *Tg.CreMYOC^Y437H^*). Under normal conditions, the mice do not express the human mutant-*MYOC* gene. Expression of Cre recombinase, however, leads to the removal of a Stop cassette and consequent expression of the DsRed-fused mutant *MYOC* (Figure 1A). A single transgene copy is inserted into a preselected intergenic and transcriptionally active genomic locus (H11) engineered with a docking site for stable and site-specific transgene integration. To confirm a site-specific knock-in of the transgene at the H11 site, we first performed PCR using primers specific to the integration site (Supplemental Figure 1A; supplemental material available online with this article; https://doi.org/10.1172/jci.insight.188710DS1, Supplemental Information), which demonstrated a stable integration of the transgene in 1 founder line. These founder mice were further bred with C57BL/6J mice, and offspring were utilized for subsequent studies. For routine genotyping, primers specific to MYOC and DsRed region were utilized, which confirmed the presence of the transgene (Supplemental Figure 1B).

### Helper Ad5-Cre induces mutant myocilin selectively in the TM.

We have previously shown that intravitreal injection of Ad5 exhibits specific tropism in the mouse TM (32–35). Therefore, we utilized Ad5 expressing Cre recombinase to induce mutant myocilin in the TM of *Tg.CreMYOC^Y437H^* mice (Supplemental Figure 2, A and B). Single intravitreal injection of Ad5-Empty or Ad5-Cre (2 × 10^7^ pfu/eye) was performed in 3-month-old *Tg.CreMYOC^Y437H^* mice. Analysis of anterior segment cross sections from the mouse eyes at 5 weeks after injection demonstrated robust MYOC-DsRed expression in the TM (Supplemental Figure 2A) of the Ad5-Cre–injected *Tg.CreMYOC^Y437H^* mice. No MYOC-DsRed was detected in the corresponding sections from control mice, nor any other ocular tissues from the Cre-induced mice, including the retina. Western blot analysis of the iridocorneal angle, retina, and sclera clearly demonstrated that mutant myocilin was selectively induced in the TM tissue of Ad5-Cre–injected *Tg.CreMYOC^Y437H^* mice, 5 weeks after the injection (Supplemental Figure 2B). Slit-lamp imaging revealed moderate ocular inflammation after Ad5-Cre injections (Supplemental Figure 2C). To reduce the ocular inflammation, we next utilized helper Ad5 (HAd5), which lacks most of the viral sequences except the *cis*-acting elements essential for viral replication and packaging. HAd5 is known to exhibit minimal immunogenicity while maintaining robust tropism for the tissue of interest (36). A single intravitreal injection of HAd5 expressing Cre or empty cassette was performed in 3-month-old *Tg.CreMYOC^Y437H^* mice. Slit-lamp imaging demonstrated no visible signs of ocular inflammation in HAd5-Empty or HAd5-Cre–injected *Tg.CreMYOC^Y437H^* mice (Figure 1B). First, we examined whether HAd5-expressing Cre exhibits functional activity in mouse TM, using fluorescence-reporter mT/mG mice. These mice express tdTomato in all tissues (red fluorescence); expression of Cre induces conversion of tdTomato to GFP (green fluorescence) (37). We performed intravitreal injection of HAd5-Empty or HAd5-Cre, and GFP/tdTomato expression was examined using confocal imaging of anterior-segment cross sections (Figure 1C and Supplemental Figure 3). Compared with empty-injected mT/mG mice, which only expressed tdTomato, Cre-injected mice exhibited GFP expression selectively in TM cells, and conversion efficiency was nearly 90% (Supplemental Figure 3). These data indicate that a single intravitreal injection of HAd5-Cre was highly efficient in transducing the mouse TM.

Next, we investigated whether HAd5-Cre induces mutant myocilin in the TM. A single intravitreal injection of HAd5-Cre or HAd5-Empty (2 × 10^7^ pfu/eye) was performed in 2-month-old *Tg.CreMYOC^Y437H^* mice, and MYOC-DsRed expression in various ocular tissues was evaluated (Figure 1, D–F). Whole mount anterior segment imaging demonstrated mutant-MYOC expression throughout the TM of Cre-injected *Tg.CreMYOC^Y437H^* mice (Figure 1D). Western blot analysis of the anterior segment (AS), retina, and choroid-sclera (CS) tissue lysates demonstrated the presence of MYOC-DsRed protein in the AS but not in the retina or CS of Cre^+^*Tg.CreMYOC^Y437H^* mice (Figure 1E). MYOC-DsRed protein was detected at approximately 75 kDa due to the DsRed tag on mutant MYOC (Figure 1E). We also detected higher molecular weight bands for MYOC, which likely represent heteromeric complexes of MYOC, as described previously (38, 39). Although endogenous myocilin was detected at 50 kDa in all ocular tissues, MYOC-DsRed (75 kDa) was only detected in the AS tissue lysates of Cre^+^*Tg.CreMYOC^Y437H^* mice, and no mutant-MYOC protein was detected in the retina and CS of Cre^+^ *Tg.CreMYOC^Y437H^* mice. Notably, we observed that endogenous myocilin protein was increased in the lysates of iridocorneal-angle tissue of Cre-injected *Tg.CreMYOC^Y437H^* mice compared with controls. Quantitative PCR (qPCR) analysis of anterior-segment tissues using primers specific to mutant MYOC confirmed the presence of MYOC mRNA in Cre-induced *Tg.CreMYOC^Y437H^* mice (Supplemental Figure 4A). We also observed that expression of mutant *MYOC* induced a 3-fold increase in endogenous *Myoc* in the AS of Cre^+^*Tg.CreMYOC^Y437H^* mice (Supplemental Figure 4B). Confocal imaging of anterior-segment cross-sections revealed a robust and selective induction of mutant MYOC in the TM of *Tg.CreMYOC^Y437H^* mice (Figure 1F). Immunostaining for α-smooth muscle actin (α-SMA), which predominantly labels the TM and ciliary muscle, revealed a strong colocalization of mutant MYOC with α-SMA in the TM region of Cre-injected *Tg.CreMYOC^Y437H^* mice (Supplemental Figure 5). These data indicate that HAd5-Cre selectively induces mutant MYOC in the TM of *Tg.CreMYOC^Y437H^* mice.

Since mutant *MYOC* is driven by the CAG promoter, which can lead to overexpression of mutant *MYOC*, we next compared the mRNA transcript of mutant *MYOC* with endogenous *Myoc* in the TM region using RNA scope (Figure 1G and Supplemental Figure 6). These data also reveal that no mRNA transcript for mutant *MYOC* was detected in Cre^–^*Tg.CreMYOC^Y437H^* mice, while abundant endogenous *Myoc* was detected in the TM region. Importantly, Cre^+^*Tg.CreMYOC^Y437H^* mice displayed the presence of mutant *MYOC* transcript selectively in the TM region. Notably, total transcript measurements (per TM cell) revealed a significant upregulation of endogenous *Myoc* expression in the TM of Cre^+^*Tg.CreMYOC^Y437H^* mice compared with Cre^–^*Tg.CreMYOC^Y437H^* mice (Supplemental Figure 6). These data indicate that mutant MYOC expression induces endogenous *Myoc* in the TM. Moreover, the expression of the mutant *MYOC* transcript in Cre-injected eyes is similar to that of the *Myoc* in Cre^–^*Tg.CreMYOC^Y437H^* mice. Together, these data establish that HAd5-Cre selectively induces mutant myocilin in the TM of *Tg.CreMYOC^Y437H^* mice at a level similar to endogenous *Myoc* without causing ocular inflammation.

### Expression of mutant MYOC reduces TM outflow and elevates IOP significantly in HAd5-Cre–injected Tg.CreMYOC^Y437H^ mice.

**Figure 2:**
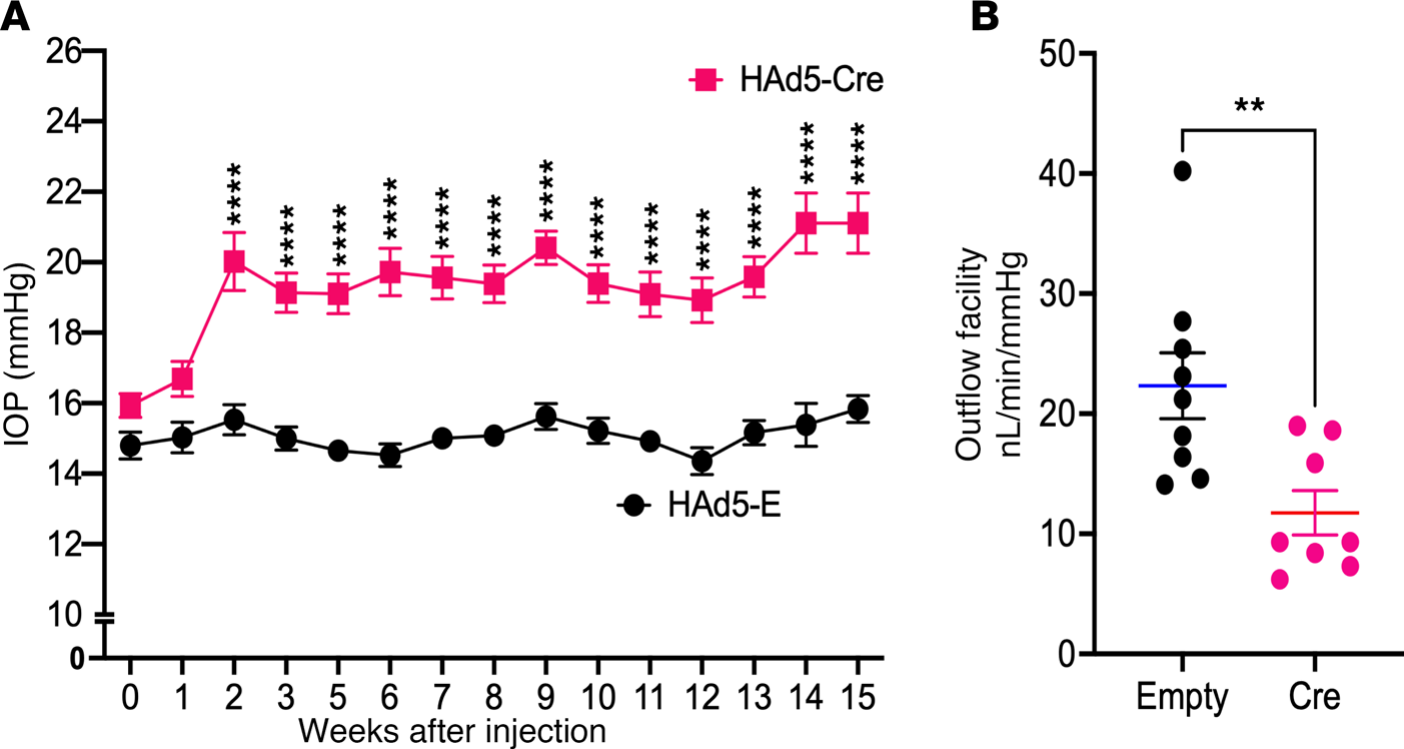
Intravitreal administration of HAd5-Cre elevates IOP and decreases outflow facility in *Tg.CreMYOC^Y437H^* mice. Three-month-old *Tg.CreMYOC^Y437H^* mice received a single intravitreal injection of either HAd5-Empty or HAd5-Cre in both eyes. (**A**) Weekly IOP measurements demonstrated significant and sustained IOP elevation in Cre-injected *Tg.CreMYOC^Y437H^* mice compared with HAd5-Empty–injected mice (*n* = 14 in Empty, and *n* = 18 in Cre-injected group; analyzed by 2-way ANOVA with multiple comparisons, ****P < 0.0001). (**B**) Outflow facility measurements showed a significant reduction in outflow facility 5 weeks post HAd5-Cre injection of *Tg.CreMYOC^Y437H^* mice (*n* = 8) compared with HAd5-Empty–injected mice (*n* = 9) (unpaired *t* test, 2-tailed, mean ± SEM, **P < 0.0072).

Three-month-old *Tg.CreMYOC^Y437H^* mice were injected intravitreally with HAd5-Empty or Cre, and IOPs were monitored weekly. Starting from 2 weeks after injection, Cre-treated *Tg.CreMYOC^Y437H^* mice exhibited significantly higher and sustained IOP compared with empty-injected *Tg.CreMYOC^Y437H^* mice (Figure 2A). Independent IOP measurements in conscious mice (without anesthesia) confirmed a pronounced and significant IOP elevation in HAd5-Cre–injected *Tg.CreMYOC^Y437H^* mice (Supplemental Figure 7). Measurement of the outflow facility using the constant flow infusion method displayed a significantly reduced outflow facility 5 weeks after HAd5-Cre injection in *Tg.CreMYOC^Y437H^* mice compared with the control group (11.75 nL/min/mmHg versus 22.32 nL/min/mmHg in Cre^+^ versus Cre^–^ *Tg.CreMYOC^Y437H^* mice) (Figure 2B). These findings demonstrate that HAd5-Cre induces significant and sustained IOP elevation due to reduced AH outflow facility from the TM in *Tg.CreMYOC^Y437H^* mice.

### Mutant myocilin induces ultrastructural and biochemical changes in the TM.

**Figure 3:**
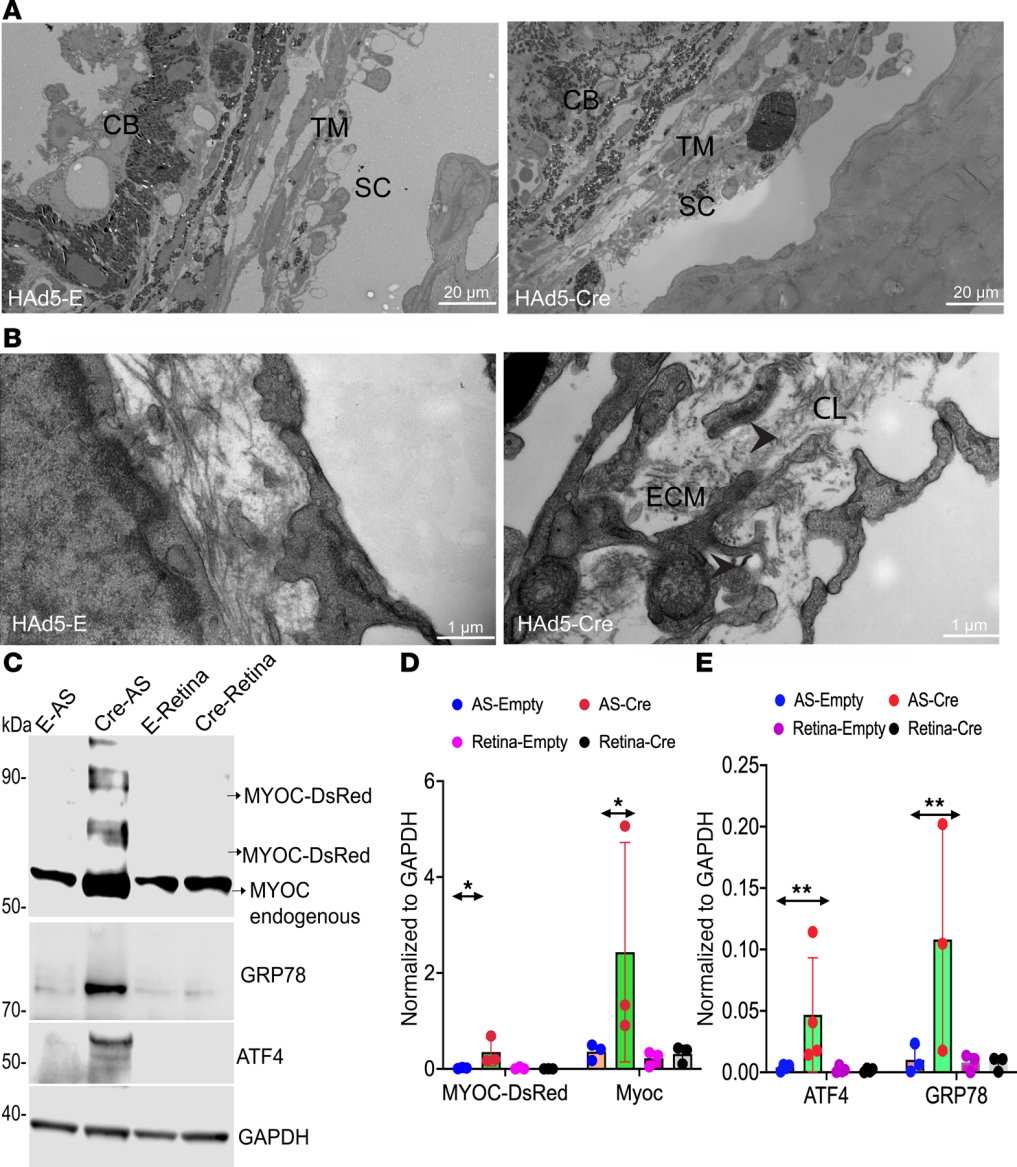
Mutant-MYOC–induced ocular hypertension is associated with ultrastructural and biochemical changes in the TM. (**A** and **B**) Representative low-magnification (**A**) and high-magnification (scale bars: 20 μm) (**B**) TEM images of *Tg.CreMYOC^Y437H^* mice 8 weeks after injection of HAd5-Empty or HAd5-Cre, showing the presence of loosely bound collagen fibers, ECM deposition, and loss of TM integrity in the juxtacanalicular-connective-tissue (JCT) region of Cre-injected *Tg.CreMYOC^Y437H^* mice (*n* = 4 in each group) (TM, trabecular meshwork; CB, ciliary body; SC, Schlemm’s canal; CL, collagen fibers; ECM, extra cellular matrix). Scale bars: 1 μm. (**C** and **D**) Western blot and densitometric analyses showing that mutant MYOC induces ER stress in the anterior-segment tissue lysates of Cre-injected *Tg.CreMYOC^Y437H^* mice (*n* = 3). (**E**) HAd5-Empty; AS, anterior segment. Two-way ANOVA with multiple comparisons (**P = 0.0053, *P = 0.0216).

In POAG, increased outflow resistance is associated with ultrastructural and biochemical changes in the TM, including increased extracellular matrix (ECM) deposition, actin-cytoskeletal changes, and induction of ER stress (11, 15, 40, 41). Next, we investigated whether mutant myocilin leads to morphological changes in the TM of *Tg.CreMYOC^Y437H^* mice. H&E staining demonstrated open-angle and no noticeable morphological changes in the anterior-chamber structures of Cre^+^*Tg.CreMYOC^Y437H^* mice, 5 weeks after injection (Supplemental Figure 8). We next performed transmission electron microscopy (TEM) to examine ultrastructural changes in the TM. Low-magnification TEM analysis demonstrated that the iridocorneal angle is open in both empty- and Cre-injected *Tg.CreMYOC^Y437H^* mice (Figure 3A). Higher-magnification TEM images revealed loosely bound collagen fibers, ECM deposition, and disrupted TM integrity in Cre^+^ *Tg.CreMYOC^Y437H^* mice compared with Cre^–^ *Tg.CreMYOC^Y437H^* mice (Figure 3B). We further confirmed these findings using immunostaining. AS were immunostained with antibodies for fibronectin (FN) and actin (Supplemental Figure 9A). FN and actin significantly increased in the TM region of Cre-injected *Tg.CreMYOC^Y437H^* mice (Supplemental Figure 9, B and C). Previous studies have shown that mutant-MYOC expression in the TM induces ER stress (29, 42–45). Therefore, we examined whether mutant-MYOC expression induces ER stress markers in the TM of Cre-induced *Tg.CreMYOC^Y437H^* mice (Figure 3C). Immunostaining (Supplemental Figure 10) and Western blot analysis (Figure 3, C–E) demonstrated significantly increased ER stress markers, including GRP78, ATF4, and CHOP selectively in the TM of Cre-injected *Tg.CreMYOC^Y437H^* mice. Together, these findings indicate that the expression of mutant myocilin induces ultrastructural and biochemical changes in the TM, leading to its dysfunction and IOP elevation in *Tg.CreMYOC^Y437H^* mice.

### Mutant-MYOC–induced sustained IOP elevation leads to functional and structural loss of RGCs.

**Figure 4:**
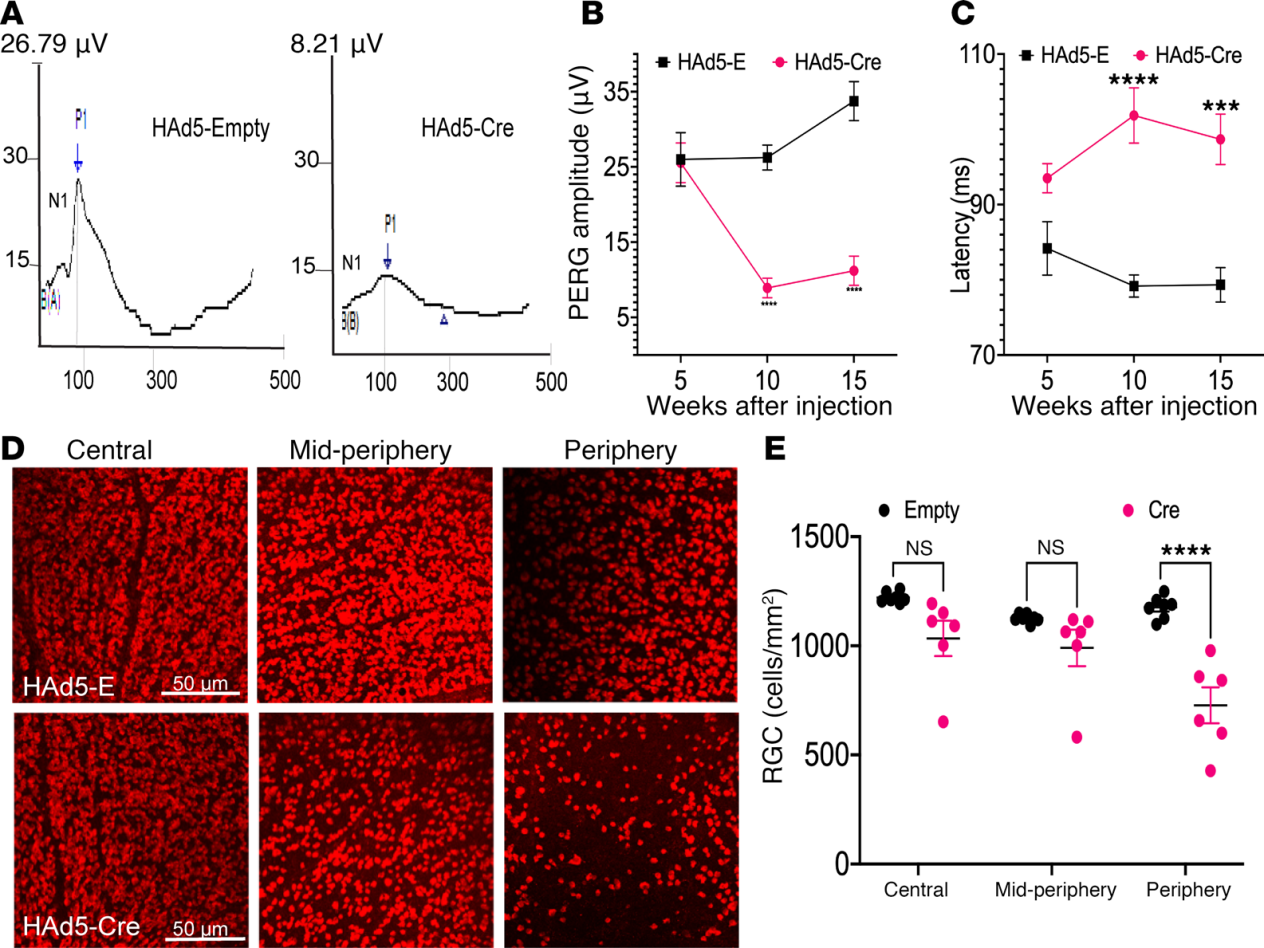
Sustained IOP elevation leads to functional and structural loss of RGCs in Cre-injected *Tg.CreMYOC^Y437H^* mice. Three- to 6-month-old *Tg.CreMYOC^Y437H^* mice were injected intravitreally with HAd5-Empty or HAd5-Cre in both eyes, and IOP was monitored weekly to ensure IOP elevation. PERG was performed at 5, 10, and 15 weeks after treatment to assess the function of the RGCs. (**A**–**C**) A representative PERG graph (**A**) and its analysis (**B** and **C**) demonstrated significantly reduced PERG amplitude (**B**) and increased latency (**C**) starting from 10 weeks post Cre-injection, indicating functional loss of RGCs in Cre^+^-*Tg.CreMYOC^Y437H^* mice (*n* = 6 in HAd5-Empty, and *n* = 6 in HAd5-Cre), 2-way ANOVA with multiple comparisons (***P = 0.0001, ****P < 0.0001). (**D** and **E**) RGC loss was further analyzed by staining the whole-mount retina with RBPMS antibody. A representative image of RBPMS staining of different regions of the retina (**D**) and its analyses (**E**) revealed a significant loss (33%) of RGCs in *Tg.CreMYOC^Y437H^* mice 15 weeks after HAd5-Cre–injection compared with control mice injected with HAd5-Empty (*n* = 7 for HAd5-Empty, and *n* = 6 for HAd5-Cre). Two-way ANOVA with multiple comparisons (****P < 0.0001). Scale bars: 50 μm.

We next evaluated whether sustained IOP elevation induced by mutant MYOC is sufficient to cause functional and structural loss of RGCs. To assess the functional loss of RGCs, we performed pattern electroretinogram (PERG) 5, 10, and 15 weeks after injection (Figure 4, A–C). Representative PERG graphs and their analysis demonstrated no significant effect on PERG at 5 weeks, but significantly reduced PERG amplitudes and increased latencies were observed at 10 and 15 weeks after Cre injection of *Tg.CreMYOC^Y437H^* mice (Figure 4, A–C). To determine the structural loss of RGCs, we next performed whole-mount retina staining with RNA binding protein with multiple splicing (RBPMS) antibody (Figure 4, D and E). As shown in representative RBPMS images, Cre-injected *Tg.CreMYOC^Y437H^* mice exhibited moderate loss of RGCs in the peripheral retina, 15 weeks after injection (Figure 4D). RGC counting further confirmed significantly reduced RGCs in the periphery of Cre-injected *Tg.CreMYOC^Y437H^* mice compared with controls at 15 weeks after injection (Figure 4E). Overall, there was a 33% loss of RGCs in the peripheral retina of Cre-injected *Tg.CreMYOC^Y437H^* mice compared with controls. We did not observe RGC loss at 10 weeks after Cre injection in *Tg.CreMYOC^Y437H^* mice (Supplemental Figure 11). These data indicate that sustained IOP elevation induced by mutant myocilin leads to functional and structural loss of RGCs in *Tg.CreMYOC^Y437H^* mice.

### Mutant-MYOC–induced sustained-IOP elevation leads to optic nerve degeneration in Tg.CreMYOC^Y437H^ mice.

**Figure 5:**
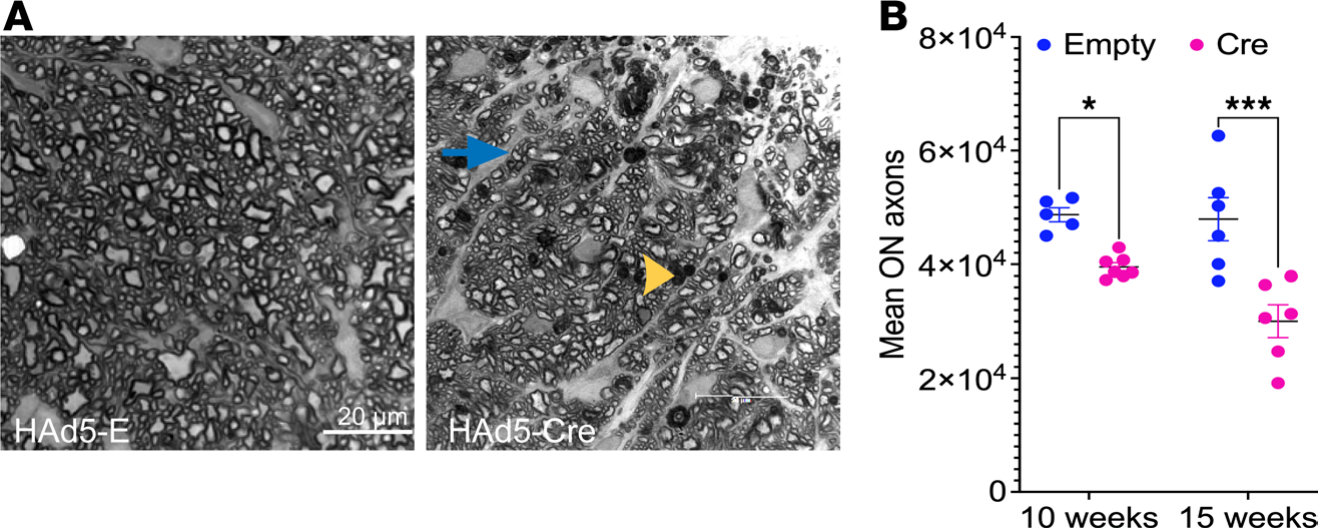
Sustained IOP elevation leads to optic nerve degeneration in Cre-injected *Tg.CreMYOC^Y437H^* mice. Optic nerves were subjected to PPD staining to assess optic-nerve degeneration. (**A**) Representative images of PPD-stained optic nerves showing mild axonal degeneration as evident from darkly stained axons (yellow arrowhead) and the presence of glial scar formation (blue arrow) in Cre-injected *Tg.CreMYOC^Y437H^* mice. Scale bars: 20 μm. (**B**) the mean axonal counts show a significant loss of ON axons (20% at 10 weeks and 45% at 15 weeks after injection in Cre-induced *Tg.CreMYOC^Y437H^* mice (*P = 0.0351 for 10 weeks after injection, *n* = 5 for Empty, and *n* = 7 for Cre; ***P = 0.0001 for 15 weeks after injection, *n* = 6 for Empty, and *n* = 6 for Cre). Two-way ANOVA with multiple comparisons.

We investigated whether sustained IOP elevation induced by mutant myocilin leads to optic nerve (ON) degeneration using paraphenylene diamine (PPD) staining (Figure 5A). Representative images of PPD-stained ON from Cre-injected *Tg.CreMYOC^Y437H^* mice revealed ON degeneration, as evident from darkly stained axons, active gliosis, and glial scar formation. Approximately 20% and 45% axonal loss was observed in Cre-injected mice at 10 and 15 weeks, respectively, compared with empty-injected *Tg.CreMYOC^Y437H^* mice (Figure 5B). To further confirm these findings, we performed GFAP immunostaining on retinal cross-sections from Cre-injected *Tg.CreMYOC^Y437H^* mice (Supplemental Figure 12A). Immunostaining for GFAP revealed a prominent increase in GFAP reactivity in the ONH region, suggesting axonal injury. Axonal degeneration was associated with a decreased neuronal marker, Tuj1, and increased mitochondrial accumulation (TOM20) in the ONH region (Supplemental Figure 12B). These data establish that sustained-IOP elevation induces ON degeneration and gliosis in the ONH of Cre-injected *Tg.CreMYOC^Y437H^* mice.

### Impaired axonal transport at the ONH precedes neuronal loss in Tg.CreMYOC^Y437H^ mice.

**Figure 6:**
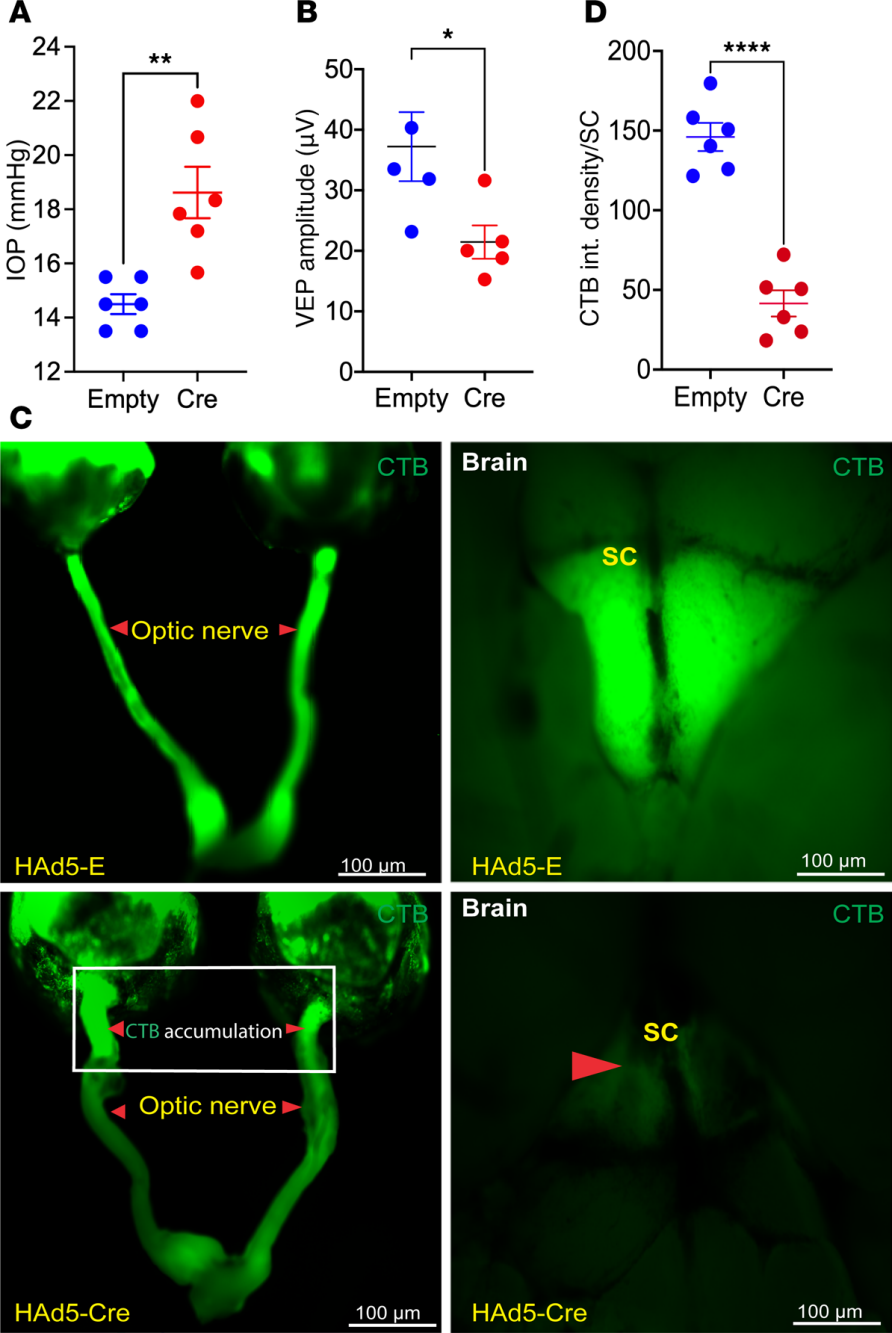
Anterograde transport deficits precede RGC degeneration in *Tg.CreMYOC^Y437H^* mice. Fifteen-month-old *Tg.CreMYOC^Y437H^* mice were injected intravitreally with HAd5-Empty or HAd5-Cre, and glaucoma phenotypes and anterograde axonal-transport mechanisms were investigated. (**A**) IOP measurement revealed significant and sustained IOP elevation at 6 weeks post-injection (*n* = 6; unpaired *t* test; *P* = 0.0058). (**B**) VEP measurements demonstrated a significant loss of postretinal visual pathway function at 7 weeks after Cre injection in *Tg.CreMYOC^Y437H^* mice (*n* = 5; unpaired *t* test; *P* = 0.0374). (**C** and **D**) Mutant-MYOC–induced ocular hypertension leads to axonal transport deficits in Cre-injected *Tg.CreMYOC^Y437H^* mice. Representative images of CTB fluorescence (**C**) and its analysis (**D**) at 7 weeks after injection of HAd5-Empty or HAd5-Cre in *Tg.CreMYOC^Y437H^* mice. Empty-injected *Tg.CreMYOC^Y437H^* mice exhibited an uninterrupted transport of CTB along the entire length of the optic nerve to the SC. However, CTB transport was blocked significantly at the ONH region, and no CTB was detected in the SC at 7 weeks after Cre injection in *Tg.CreMYOC^Y437H^* mice (*n* = 6 in each group). Scale bars: 100 μm. Small arrowheads indicate optic nerve, and large arrowhead indicates superior colliculus (SC).

Since *Tg.CreMYOC^Y437H^* mice exhibit well-defined timelines for RGC-axon loss; we further sought to understand the early pathogenic events preceding neuronal loss. Studies of RGC loss and ON degeneration suggested that axonal changes at the ONH may be the first site of damage in ocular hypertensive *Tg.CreMYOC^Y437H^* mice. We, therefore, hypothesize that IOP-induced neurodegenerative changes in the ONH precede neuronal loss. Since *Tg.CreMYOC^Y437H^* mice did not show significant structural loss of RGCs at 10 weeks after Cre injection (Supplemental Figure 11), we examined whether axonal dysfunction occurred at the ONH prior to RGC loss at 7 weeks after Cre injection. To test this, 15-month-old *Tg.CreMYOC^Y437H^* mice were injected intracamerally with HAd5-Empty or Cre, and IOPs were monitored. IOP measurements confirmed a significant IOP elevation 6 weeks after Cre injection of *Tg.CreMYOC^Y437H^* mice (Figure 6A). PERG, which measures the function of RGC soma, revealed no significant functional loss of RGC soma at 5 weeks after Cre injection of *Tg.CreMYOC^Y437H^* mice (Figure 4, A–C). Whole-mount retinal staining with RBPMS revealed no significant loss of RGCs at 10 weeks after Cre injection of *Tg.CreMYOC^Y437H^* mice (Supplemental Figure 11). Notably, visual evoked potential (VEP), which measures the postretinal function of the visual system — including ON and visual centers of the brain — demonstrated a significant loss of VEP amplitudes at 7 weeks, suggesting axonal dysfunction in Cre^+^*Tg.CreMYOC^Y437H^* mice (Figure 6B). To understand whether axonal dysfunction occurs at early stages of neuronal loss due to sustained IOP, we examined the anterograde transport of fluorescently labeled cholera toxin B (CTB), as described previously (46). At 7 weeks after injection, HAd5-Empty or HAd5-Cre–injected *Tg.CreMYOC^Y437H^* mice were intravitreally injected with green fluorescently tagged CTB dye (Figure 6C). Forty-eight hours after injections, anterograde transport of CTB through the ON and superior colliculus (SC) was monitored via fluorescence microscopy. As expected, CTB was transported to the SC in HAd5-Empty–injected *Tg.CreMYOC^Y437H^* mice. However, CTB transport was completely blocked at the ONH, and no CTB was observed in the SC of Cre-injected *Tg.CreMYOC^Y437H^* mice. Measurement of CTB fluorescence in the SC demonstrated a significant (~72%) loss of CTB in the SC of Cre-injected *Tg.CreMYOC^Y437H^* mice (Figure 6D). Previous studies have shown that axonal transport deficits are age dependent (47). Since we observed significant axonal transport in 15-month-old Cre^+^*Tg.CreMYOC^Y437H^* mice, we next explored whether axonal transport deficits are observed in younger Cre^+^*Tg.CreMYOC^Y437H^* mice. Four-month-old *Tg.CreMYOC^Y437H^* mice were intravitreally injected with HAd5-Empty or HAd5-Cre; at 7 weeks after injection, ocular hypertensive eyes were analyzed for CTB transport (Supplemental Figure 13, A and C). Similar to 15-month-old mice, CTB transport was blocked at the ONH, and a significant reduction (65%) in CTB transport to the SC was observed in 4-month-old *Tg.CreMYOC^Y437H^* mice (Supplemental Figure 13, B and C). Altogether, these data indicate that OHT impairs axonal transport by blockage at the ONH prior to RGC loss in Cre^+^*Tg.CreMYOC^Y437H^* mice in an age-independent manner.

### Ultrastructural changes in RGC axons are associated with impaired axonal transport in ocular hypertensive Cre^+^Tg.CreMYOC^Y437H^ mice.

**Figure 7:**
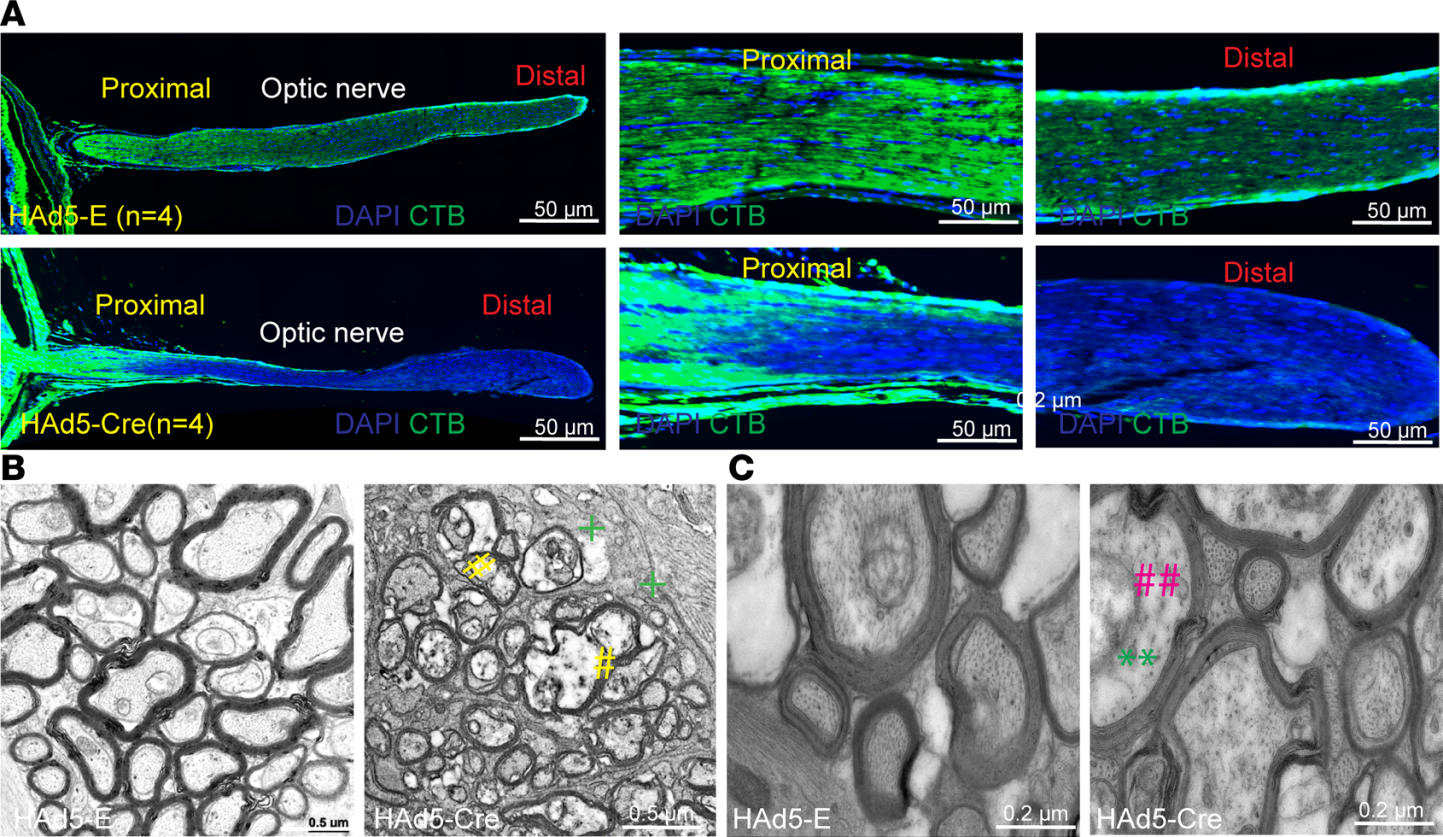
Decreased microtubules and neurofilament are associated with axonal transport deficits in the ONH region. (**A**) Whole eyes along with the ON from CTB-injected *Tg.CreMYOC^Y437H^* mice were sectioned to image CTB transport along the ON. Retinal cross-sections demonstrated blockage of CTB transport in the proximal region of the ON in Cre-injected *Tg.CreMYOC^Y437H^* mice (*n* = 3). Scale bars: 50 μm. (**B** and **C**) Low-magnification (scale bars: 0.5 μm) (**B**) and high-magnification (**C**) TEM analysis of optic nerves from *Tg.CreMYOC^Y437H^* mice injected with HAd5-Empty or HAd5-Cre, demonstrating cytoskeleton degeneration, organelle accumulation, and the presence of glial scar prior to RGC degeneration at 7 weeks after Cre injection (*n* = 4). #, organelle accumulation; +, glial scar; **, neurofilament; ##, microtubule. Scale bars: 0.2 μm.

To further gain molecular insights about impaired axonal transport, we analyzed CTB transport in a cross-section of the retina, along with the ON. CTB transport was blocked in the proximal region of the ON in Cre-injected *Tg.CreMYOC^Y437H^* mice (Figure 7A). TEM analysis of the ON 8 weeks after Cre-injection demonstrated ultrastructural changes, including loss of neurofilament (NF) and microtubule structures (Figure 7, B and C). The presence of myelin around axons in Cre-injected mice indicated intact axons. However, the accumulation of intracellular materials and loss of microtubules and NFs suggest that these ultrastructural changes in ON axons precede axonal degeneration. To confirm the TEM findings, we performed immunostaining on retinal cross-sections obtained from Cre^–^ and Cre^+^ *Tg.CreMYOC^Y437H^* mice 7 weeks after injection (Supplemental Figure 14). Cre^+^*Tg.CreMYOC^Y437H^* mice exhibited a dramatic increase in GFAP reactivity, decreased microtubules (Tuj1), and NF loss in the ONH region compared with Cre^–^*Tg.CreMYOC^Y437H^* mice. Importantly, we observed increased mitochondrial accumulation in RGC soma (Supplemental Figure 14) and axons at the ONH region in Cre^+^*Tg.CreMYOC^Y437H^* mice, 12 weeks after injection (Supplemental Figure 12). Together, our studies suggest that the loss of microtubules and NFs, which are required for axonal transport, is associated with impaired axonal transport, leading to the accumulation of defective organelles and causing axonal dysfunction.

### Axonal transport remains intact in ocular-hypertensive mice injected with MBs despite significant RGC loss.

**Figure 8:**
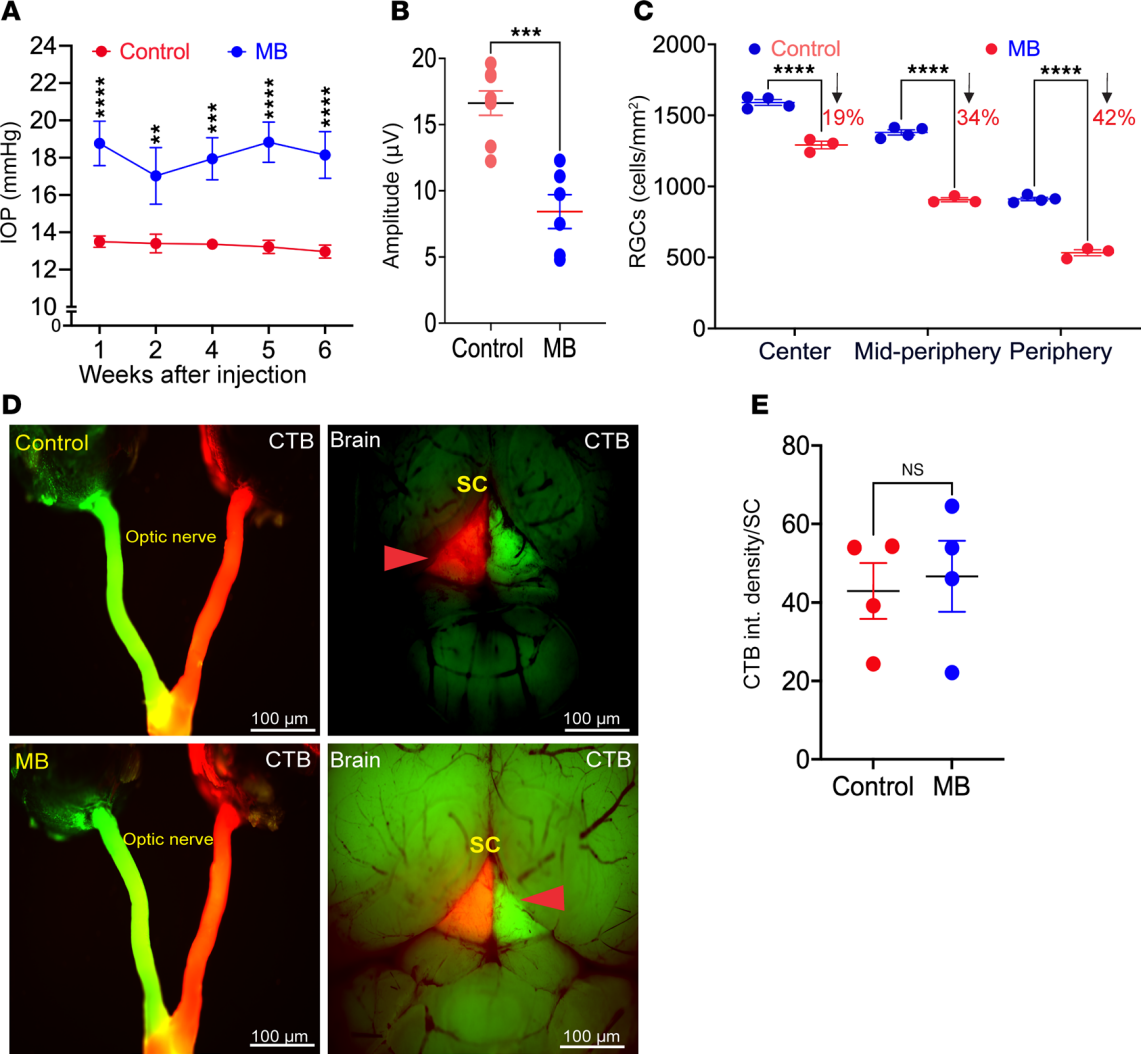
Anterograde axonal transport remains intact despite RGC degeneration in ocular hypertensive mice injected with microbeads. Four-month-old C57BL/6 mice were injected intracamerally with PBS or microbeads (MB). Glaucoma phenotypes and anterograde axonal transport mechanisms were investigated. (**A**) IOP measurements revealed significant and sustained IOP elevation in MB-injected mice starting from the first week of injection. Note that only ~50% of eyes injected with MB exhibited the expected IOP elevation; i.e., ≥ 4 mm Hg. The graph only includes eyes that showed sustained IOP elevation of 4 mm Hg or more. (*n* = 10 for control, *n* = 7 MB; analyzed by 2-way ANOVA with multiple comparisons; ****P < 0.0001). (**B**) PERG measurements demonstrated a significant functional loss of RGCs at 6 weeks after MB injection (*n* = 8 control, *n* = 6 MB; unpaired *t* test, 2-tailed, mean ± SEM, ***P = 0.0002). (**C**) Analysis of RGCs by RBPMS staining of retinal whole mounts revealed a significant loss of RGCs at 6 weeks after MB injection (*n* = 4 for control, *n* = 3 MB; analyzed by 2-WAY ANOVA with multiple comparisons, ****P < 0.0001). (**D** and **E**) Representative images of CTB fluorescence in the optic nerve and SC, and analysis of CTB fluorescence in ocular hypertensive mice 4 weeks after MB injection. CTB transport remained intact despite significant RGC loss in MB-injected mice (*n* = 4 in each group; unpaired *t* test). Scale bars: 100 μm.

To determine whether impaired axonal transport is a unique and early pathological hallmark of Cre^+^*Tg.CreMYOC^Y437H^* mice, we compared axonal transport mechanisms in a mouse model of MB-induced OHT. MB-induced OHT is a widely utilized model to study glaucomatous neurodegeneration (21, 48). Four-month-old C57BL/6J mice were intracamerally injected with magnetic MBs, which block TM outflow, elevating IOP, as described previously (47, 49). Weekly IOP measurements demonstrated that about 60% of MB-injected eyes exhibited significant IOP elevation (≥4 mmHg) (Figure 8A). The eyes that did not display IOP elevation likely reflect a technical failure to keep the MBs in the outflow pathway. The ocular hypertensive eyes (IOP elevation > 4 mmHg) at 6 weeks after MB injection were selected for functional and structural analysis of the RGCs, using PERG measurements and RBPMS staining, respectively. PERG (Figure 8B) and RBPMS staining (Supplemental Figure 15 and Figure 8C) demonstrated significant structural and functional loss of RGCs in the MB-injected eyes. GFAP staining (Supplemental Figure 15A) demonstrated that a modest increase in astrocyte labeling is associated with RGC loss in the periphery of the retina of MB-injected mice. However, no significant change in GFAP reactivity was observed in the midperiphery and ONH region of MB-injected eyes. Moreover, immunostaining for Tuj1 and NFs revealed no significant change in the ONH region of MB-injected eyes (Supplemental Figure 15B). Next, we investigated whether ocular-hypertensive MB-injected mice exhibit axonal-transport deficits, as seen in Cre^+^*Tg.CreMYOC^Y437H^* mice. Four-month-old C57BL/6J mice were injected with PBS or MB, and IOPs were monitored (Supplemental Figure 16A). PERG measurements in ocular-hypertensive eyes at 3 weeks after MB injection revealed a significant functional loss of RGCs in MB-induced OHT mice (Supplemental Figure 16B). CTB was injected intravitreally at week 4 after MB injection, and CTB transport to the SC was examined (Figure 8, D and E). Despite the significant functional loss of RGCs, we observed that CTB transport to the SC was completely intact (Figure 8, D and E). These data indicate that axonal transport persists despite significant RGC degeneration in a mouse model of MB-induced OHT.

## Discussion

In the present study, we describe the development of a new Cre-inducible mouse model of MYOC-associated POAG. We report that HAd5 drives the TM-specific expression of mutant MYOC, leading to TM dysfunction and IOP elevation. Notably, a single intravitreal injection of HAd5-Cre is sufficient to induce sustained IOP elevation, which leads to glaucomatous neurodegeneration in a highly predictable manner. The induction of mutant MYOC via Cre expression in adult mice allowed us to study early events of glaucomatous damage to the TM and IOP-induced neurodegeneration. Importantly, we show that sustained IOP elevation impairs axonal transport at the ONH prior to loss of RGC somas and axons. These mechanistic insights into early events of TM dysfunction and axonal degeneration identify valuable targets for the development of IOP-lowering and neuroprotective therapies for glaucoma.

Previously, we developed a transgenic mouse model (*Tg-MYOC^Y437H^*) by random insertion of mutant MYOC (29). That transgenic mouse model exhibited robust glaucoma phenotypes on a mixed background, and using it, we discovered the pathological role of chronic ER stress in the pathophysiology of glaucomatous TM damage (28, 29, 32). However, those mice exhibited only mild glaucoma phenotypes upon subsequent breeding onto a pure C57BL/6J background (35, 50, 51). We interpreted this diminution of phenotype to be due to the silencing of the transgene, likely in response to the toxic nature of mutant MYOC. Moreover, our previous transgenic mouse model presented several limitations, including a lack of robust glaucomatous neurodegeneration on the pure C57BL/6J background, unknown copies of mutant MYOC, and a lack of specific antibodies to distinguish human versus mouse myocilin. Development of the *Tg.CreMYOC^Y437H^* mice has overcome these key limitations. The advantages include the targeted insertion of mutant MYOC in a preselected intergenic and transcriptionally active genomic locus (H11). This approach achieves stable and site-specific transgene integration of a single copy of mutant MYOC. Also, *Tg.CreMYOC^Y437H^* mice are developed on a pure C57BL/6J background. Furthermore, DsRed is fused with the mutant *MYOC*, which enabled us to distinguish mutant *MYOC* from endogenous *Myoc*. Indeed, RNAscope analysis demonstrated that the expression levels of mutant *MYOC* are similar to those of endogenous *Myoc*. This was further documented by Western blot analysis, which demonstrated that protein levels of human mutant MYOC fused with DsRed are comparable with endogenous MYOC in Cre^–^*Tg.CreMYOC^Y437H^* mice. These data indicate that glaucoma phenotypes in *Tg.CreMYOC^Y437H^* mice are not a result of overexpression of mutant MYOC. RNAscope, qPCR, and Western blot analyses demonstrated that expression of mutant *MYOC* led to the induction of the endogenous *Myoc* gene and protein. This observation is consistent with previous findings that TM cells induce MYOC in response to various insults, including glucocorticoid treatment (52–54). Although the exact function of endogenous myocilin is unclear, previous studies have suggested its role as a stress-response factor (55). Misfolded mutant MYOC likely interacts with endogenous myocilin protein, causing its accumulation in the ER (38) and further contributing to TM dysfunction.

Our current study has a few limitations. First, we employed a viral vector to induce Cre expression in the TM. The Cre-*LoxP* recombinase system is an effective and widely used experimental tool to investigate genes of interest in tissue/cell- and/or time-specific ways (56). Cre-inducible mice are often crossed with tissue-specific Cre mouse lines to induce Cre expression. Crossing *Tg.CreMYOC^Y437H^* mice with a TM-specific Cre mouse line would be ideal to induce mutant MYOC in the TM. However, there are no TM-specific Cre mouse lines available. The global Cre mouse lines would express mutant MYOC in other cell types, including in the ciliary body and retina, which could result directly in the death of ciliary-epithelial cells or RGCs. Previous studies have shown that mutant MYOC is toxic to cells (29), and degeneration of the nonpigmented epithelium in the ciliary body was observed in older *Tg-MYOC^Y437H^* mice (51). We also observed that induction of mutant MYOC selectively in RGCs via intravitreal injection of AAV2-Cre leads to severe RGC loss within 3 weeks of injection in *Tg.CreMYOC^Y437H^* mice (data not shown). A recent study has shown that a Matrix Gla–Cre (MGP-Cre) mouse line exhibits Cre activity in the TM and other ocular cells relevant to glaucoma pathology (57). Accordingly, our future studies will focus on using the MGP-Cre mouse line to induce mutant MYOC in the TM and other ocular cells. Secondly, we did not investigate the long-term effects of mutant MYOC on TM dysfunction and neuronal loss. We expect IOP elevation to be sustained throughout the lives of the *Tg.CreMYOC^Y437H^* mice, resulting in severe neuronal loss, as observed in POAG. Thirdly, the robust glaucoma phenotype depends on successful intraocular injection to ensure Cre expression throughout the entire TM, a process that can be technically challenging and requires practice. Cre-injected *Tg.CreMYOC^Y437H^* mice that do not exhibit a robust phenotype are likely the result of incomplete MYOC expression throughout the entire TM. Although both intracameral and intravitreal injections of HAd5-Cre induce mutant myocilin in the TM to a similar degree, we find that slow perfusion of HAd5-Cre via the intracameral route is more effective in elevating IOP, but it is difficult and requires a perfusion pump.

*Tg.CreMYOC^Y437H^* mice recapitulate the pathophysiology of human POAG, including IOP elevation and reduced outflow facility due to TM dysfunction. Like POAG, we observed ultrastructural and biochemical changes associated with TM dysfunction, as evident from increased ECM deposition, actin, and ER stress in Cre-injected mice. Importantly, sustained IOP elevation leads to progressive axonal degeneration, which is associated with RGC loss. In contrast to other familiar glaucoma models, most of the *Tg.CreMYOC^Y437H^* mice develop OHT and exhibit neuronal loss. Nearly all eyes treated with Cre developed IOP elevation and glaucomatous neurodegeneration. Thus, glaucoma phenotypes in Cre-inducible *Tg.CreMYOC^Y437H^* mice are highly predictable, and the exact timeline of each phenotype can be easily tracked. Mutant MYOC is induced within a week, elevating IOP starting from 2 weeks after Cre injection, followed by axonal-transport deficits at 7 weeks after injection. Axonal loss is observed at 10 weeks, and RGC soma loss is observed at 15 weeks. These features make the *Tg.CreMYOC^Y437H^* mouse model highly attractive for studying TM dysfunction and neuronal loss in glaucoma.

Our study further demonstrates that sustained IOP elevation impairs axonal transport in the ONH prior to axonal degeneration and RGC-structural loss. Axonal-transport deficits are considered an important pathological feature of glaucomatous neurodegeneration (4, 58). Previous studies have shown that impaired axonal transport is associated with glaucomatous neurodegeneration (5, 47, 59–61). In this regard, we recently demonstrated that axonal transport persists during the initial stages of axonal degeneration in a mouse model of glucocorticoid-induced glaucoma (46). In addition, several other studies have shown that impaired axonal transport in the ONH region occurs in an inducible model of OHT (4–6, 46, 62). However, it is poorly understood whether impaired axonal transport is caused by, or is a cause of, RGC degeneration. In Cre-injected *Tg.CreMYOC^Y437H^* mice at 7 weeks, we observed a complete blockage of axonal transport without a significant loss of axons, as evident from intact myelin. TEM analysis demonstrated intracellular ultrastructural changes, including swollen axons, increased mitochondrial accumulation, and gliosis, indicating axonal dysfunction. Interestingly, we observed a significant loss of VEP, which measures postretinal visual pathways (ON and visual center of the brain) (63), while RGC function (measured by PERG) was not significantly affected until 10 weeks after injection. Together, these findings indicate that impaired axonal transport at the ONH precedes RGC soma and axonal loss. Since axonal transport is essential for RGC soma survival, including the transport of organelles, synaptic components, vesicles, and neurotrophic factors, it is likely that impaired axonal transport leads to degeneration of RGC axons and loss of RGC somas. Consistent with this interpretation, we observed increased mitochondrial accumulation in RGC somas and ON axons in Cre-injected *Tg.CreMYOC^Y437H^* mice, at 12 weeks after injection.

It is pertinent to note that, with the MB-model mice, axonal transport persisted despite significant RGC loss. A previous study had shown that significant axonal transport was observed in the MB model initially at the SC, which then progresses proximally to the ON (47). Moreover, axonal-transport deficits were only observed in elderly animals, despite young and old mice showing RGC loss. These observations suggest that impaired axonal transport is unlikely to cause RGC loss. In contrast, impaired axonal transport in *Tg.CreMYOC^Y437H^* mice occurred at the early stage of neurodegeneration, and it was observed in both young and old mice. These findings highlight differences in early pathological events in the different models, despite similar IOP elevation. In *Tg.CreMYOC^Y437H^* mice, elevated IOP may cause primary damage to the ONH resulting in loss of transport, which then proceeds to loss of RGCs. In the MB model, elevated IOP may cause direct loss of RGC somas, which then progresses to axonal degeneration and impaired axonal transport. Several lines of evidence support that ONH is the first site of injury due to mutant MYOC-induced OHT. First, significant loss of RGC axons was observed as early as 10 weeks, while RGC soma loss was observed later at 15 weeks. Second, VEP, which measures the postretinal function of the visual system (including ON and visual centers of the brain), is reduced significantly prior to RGC functional or structural loss. Third, impaired axonal transport was observed in the ONH region as early as 7 weeks after injection prior to RGC-functional loss in an age-independent manner.

Microtubules and NFs are key cytoskeleton proteins required for axonal transport; loss of these proteins is associated with glaucomatous neurodegeneration (62, 64, 65). Here, we propose that loss of microtubules and NFs at the ONH due to chronic IOP elevation is likely responsible for impaired axonal transport. TEM and immunostaining demonstrated dramatic loss of cytoskeletal proteins, including microtubules and NFs associated with impaired axonal transport. This is the first study to our knowledge showing that IOP elevation induced loss of microtubules, impairing axonal transport in the ONH prior to neuronal loss. Since both microtubules and NF assembly are regulated by autophagy (66), chronic IOP elevation may lead to compromised autophagy, which would result in the loss of NFs and microtubule assembly, accumulating defective organelles in the ONH region. Consistent with this scenario, several studies have suggested that chronic IOP elevation leads to compromised autophagy in the RGC axons (67, 68). Alternatively, the loss of microtubules, which facilitate the formation and transport of autophagic vesicles carrying defective organelles, may contribute to the accumulation of autophagic vesicles in the ONH region, including defective mitochondria, as evident in our studies. Further understanding of these early events of axonal degeneration identifies a therapeutic focus for regeneration strategies to support dying axons.

Elevated IOP has differential effects on axonal transport mechanisms in the 2 models, likely due to differences in the sites of primary insult. Unlike in *Tg.CreMYOC^Y437H^* mice, the MB-induced OHT did not alter the neuronal cytoskeleton (Tuj1 and NFs) or astrocyte reactivity in the ONH, thus allowing active axonal transport, despite functional and structural loss of RGC somas. The primary site of insult in *Tg.CreMYOC^Y437H^* mice due to OHT is the ONH, as evident from reduced axonal transport, which is associated with decreased microtubules and NFs (key players in axonal transport). It is unclear why OHT caused by the MB injection induces RGC soma loss without affecting ONH axons. It is possible that MBs exert IOP-independent effects directly on RGC somas. Our findings with the MB model are consistent with a previous study by Crish et al. (47), which demonstrated that axonal transport deficits are age dependent and not necessarily associated with OHT. These findings also suggest that the MB model does not accurately replicate human POAG, in which impaired axonal transport occurs during the early stages of neurodegeneration. It is conceivable that the remaining RGCs could compensate for the transport of CTB to the SC in the MB model. Consistent with this interpretation, Crish et al. showed that axonal transport impairment occurs in the ONH during the later stage of axonal degeneration, which is associated with substantial damage to RGC axons.

In summary, we developed a Cre-inducible mouse model of POAG that faithfully replicates all features of human POAG, including IOP elevation due to TM dysfunction and IOP-dependent glaucomatous neurodegeneration. We expect that the *Tg.CreMYOC^Y437H^* mouse model will be a valuable tool for studying the pathophysiology of glaucomatous damage to the TM and RGC axons and for identifying new targets for therapeutic development.

## Methods

Supplemental Methods are available online with this article.

### Sex as a biological variable.

Both males and females were used in the current study. Sex was not considered a biological variable.

### Animal husbandry.

Animals were housed and bred in standard 12-hour light/12-hour dark conditions. They were fed standard chow ad libitum and housed in cages with dry bedding. Two- to 3-month-old C57BL/6J mice (including both males and females) were obtained from The Jackson Laboratory. mT/mG mice were obtained from The Jackson Laboratory (stock no. 007576) and bred as described previously (37). *Tg.CreMYOC^Y437H^* transgenic mice on a pure C57BL/6J background were generated in the lab as described below. Mice were euthanized via inhalation of carbon dioxide, followed by cervical dislocation, as described previously (32).

### Generation of Tg.CreMYOC^Y437H^ mice.

*Tg.CreMYOC^Y437H^* mice were generated using the TARGATT site-specific transgenic technology (Applied Stem Cell) according to the manufacturer’s protocol (30). Briefly, the cDNA for MYOC with the Y437H mutation was subcloned into a TARGATT plasmid (AST-3050, Applied Stem Cell) that contains the attB recombination site. The expression cassette contained a STOP signal flanked with a loxP site in the front of the Y437H MYOC cDNA under the control of the CAG promoter. Additionally, the DsRed tag was fused to the C-terminus of the human mutant myocilin. A mixture of the donor plasmid (containing the attB sites and the transgene) and the ΦC31 mRNA was injected into the pronuclei of H11P3-C57BL/6J mouse embryos. The genomic DNA of a founder was analyzed using primer pairs for the transgene and the H11 locus to verify site-specific insertion. The primer set AST 2005 (Applied Stem Cell) was utilized to detect H11 site-specific integration. Transgene-positive mice were bred with C57BL/6J mice, and heterozygous mice were utilized for experiments. The following primers were utilized to detect the transgene during regular genotyping. Forward primer: 5′-CTCAGCAGATGCTACCGTCA-3′; reverse primer: 5′-GCACCTTGAAGCGCATGAA-3′.

### Cre recombinase induction by viral vectors.

Ad5-Cre or HAd5-Cre under the control of the CMV promoter were utilized to selectively induce Cre activity in the TM. Ad5 and HAd5, each expressing empty cassettes, were utilized as controls. These vectors were purchased from the Viral Core Facility at the University of Iowa. Mice were anesthetized using 2.5% isoflurane plus 100% oxygen. A single intravitreal injection of Ad5 or HAd5, each expressing Cre or empty cassette (2 × 10^7^ pfu/eye), was performed as described previously (31, 32, 69). Male and female *Tg.CreMYOC^Y437H^* mice (3–6 months old) were used in all experiments, except for axonal transport experiments, where 15-month-old *Tg.CreMYOC^Y437H^* mice were utilized. For CTB (Invitrogen, C22841) injections in 15-month-old *Tg.CreMYOC^Y437H^* mice, we performed intracameral injection of HAd5-Empty or Cre, as described previously (46). For all other experiments, intravitreal injection was utilized.

### Antibodies and reagents.

The following antibodies were used in the current study. Myocilin monoclonal antibody (60357-1-Ig, Proteintech); DsRed antibody (600-401-379, Rockland); KDEL (Ab12223, Abcam); ATF4 (SC-200, Santa Cruz Biotechnology); CHOP (13172, Novus Biologicals); GRP78 (ab21685, Abcam); GRP94 (11402, Santa Cruz Biotechnology); RBPMS (118619, Gene Tex); GAPDH (3683, Cell Signaling Technology); TOM20 (11802-1-Ap, Proteintech); TUJ1(GTX130245, Genetex); GFAP (ab4674-1001, Abcam); FN (AB2413-1001); neurofilament heavy (NF-H) (80170l, BioLegend); Phalloidin stain for actin (12956, Cell Signaling Technology); and anti–α-SMA (ab5694, Abcam).

### Real-time PCR.

The AS of Cre^–^- and Cre^+^-*Tg.CreMYOC^Y437H^* mice were submerged in RNAprotect Cell Reagent (76526, QIAGEN). Subsequently, the AS was pelleted by centrifugation for 5 minutes at 600*g* and then subjected to total RNA extraction using a RNeasy Mini Kit (QIAGEN). Following RNA extraction, real-time PCR was carried out using the SYBR Green Quantitative qPCR Kit (QR0100, Sigma Aldrich) with the following primers: *MYOC*: forward primer, 5′-TGACTTGGCTGTGGATGAAG-3′; reverse primer: 5′-TTGTCTCCCAGGTTTGTTCG-3′. *Myoc*: forward primer 5′-GCC ATC CAA GAC CTT CAG AG-3′; reverse primer: 5′-AGA TCC CTG GTT TGG GTC TC-3′. The housekeeping gene, HPRT: forward primer, 5′-TGA CAC TGG CAA AAC AAT GCA-3′; reverse primer: 5′-GGT CCT TTT CAC CAG CAA GCT-3′.

### Western blot analysis.

Ocular tissues were carefully dissected and lysed in RIPA buffer (Thermo Fisher Scientific), as described previously (29, 35). For anterior-segment tissues, iridocorneal rings, including TM and surrounding tissues, were collected. Retina samples contained the entire retina, including the ON region. Approximately 20–30 μg of total protein was applied to each lane and separated on denaturing 4%–12% gradient polyacrylamide ready-made gels (NuPAGE Bis–Tris gels, Invitrogen) before being transferred onto PVDF membranes (MilliporeSigma). The blots were blocked with 10% nonfat dried milk for 1 hour and then incubated overnight with specific primary antibodies at 4°C on a rotating shaker. Membranes were washed 3 times with phosphate-buffered saline/Tween buffer (PBST) and were incubated with the corresponding HRP-conjugated secondary antibody for 90 minutes. Proteins were visualized on the LI-COR Odyssey Fc image system, using ECL detection reagents (Super Signal West Femto Maximum Sensitivity Substrate; Invitrogen) (31, 46). The same blot was subsequently incubated with a GAPDH antibody (Cell Signaling Technology) to document equal protein loading.

### AS flat mounts.

A whole anterior-segment flat mount was performed to image DsRed-MYOC levels in the entire TM region. Cre^–^- and Cre^+^-*Tg.CreMYOC^Y437H^* mice were enucleated, fixed with 4% paraformaldehyde (PFA), and dissected carefully to separate the anterior and posterior segments. AS were then transferred onto a slide and cut into 4 quadrants to place tissue flat on the slide. AS were mounted with a DAPI-mounting solution, and images were captured using a Keyence microscope.

### RNA scope.

Fluorescent in situ hybridization was performed using the RNAscope Multiplex Fluorescent Assay v2 (ACD Diagnostics) following modifications as described previously (70). Briefly, freshly thawed frozen histologic sections of mouse eyes were pretreated according to the manufacturer’s protocol using hydrogen peroxide and target retrieval reagents, including protease IV. Probes were then hybridized according to the protocol and then detected with TSA Plus Fluorophores fluorescein, cyanine 3, and cyanine 5. Sections were mounted with Prolong Gold Antifade (Thermo Fisher Scientific) with a coverslip for imaging and were imaged using a confocal microscope (SP8, Leica). Probes specific for *Myoc* (catalog 506401-C3) and *MYOC* (catalog 506541) transcripts were designed by the manufacturer (ACD Diagnostics).

### Immunostaining.

Enucleated eyes were fixed with 4% PFA for 3 hours. The eyes were embedded in paraffin or OCT compound (Tissue-Tek). Sections of 5 or 10 μm were cut and utilized for immunostaining, as described previously (29, 46). For paraffin-embedded samples, the slides were deparaffinized and rehydrated, followed by antigen retrieval with citrate buffer (pH 6). For OCT-embedded sections, slides were washed with PBS (Genesee Scientific, 25-508). The slides were then incubated with blocking buffer (10% goat serum [EMD Millipore S26-LITER] and 0.5% Triton-X-100 [MilliporeSigma, T8787] in PBS) for 2 hours. The slides were incubated with the primary antibody in a blocking buffer overnight. After 3 washes with PBS, the slides were incubated with the appropriate Alexa Fluor secondary antibodies (Invitrogen) for 2 hours. Following 3 final washes in PBS, the sections were mounted with a DAPI mounting solution, and images were captured using a Keyence microscope as described previously (41, 46).

### Mouse slit-lamp examination.

To evaluate ocular abnormalities and inflammation in HAd5-Empty– and HAd5-Cre–injected eyes, slit-lamp microscopy (SL-D7; Topcon) was performed as described previously (29).

### IOP measurements.

IOP measurements were conducted using the TonoLab rebound tonometer (Colonial Medical Supply) under anesthetic conditions (isoflurane [2.5%]; oxygen [0.8 L/min]), as described previously (35). All IOP assessments were performed between 10 a.m. and 2 p.m. in a masked manner. In addition, IOP measurements were replicated by another technician in a masked manner. The final IOP value was obtained by averaging 6 individual IOP measurements. Conscious IOPs were measured as described previously (71).

### Axonal transport.

To assess the axonal anterograde transport mechanism, fluorescently labeled CTB was injected intravitreally, and its transport across the ON to the SC was tracked using a fluorescence microscope, as described previously (46). Both 4- and 15-month-old *Tg.CreMYOC^Y437H^* mice were used to determine whether CTB transport blockage is age dependent, as described by a previous study (47). Seven weeks after either HAd5-Empty or HAd5-Cre injection, *Tg.CreMYOC^Y437H^* mice were anesthetized using isoflurane and an intravitreal injection of 3 μL of 0.1% CTB (reconstituted in PBS), conjugated with either Alexa Fluor 555 or Alexa Fluor 480 (Invitrogen), was performed. Forty-eight hours later, mice were euthanized, and eyes and whole brains were fixed in 4% PFA at 4°C for 12 hours. Subsequently, tissues were washed with PBS, cryoprotected in a sucrose gradient (10%–30%), embedded in OCT compound, and cryosectioned at a thickness of 10 μm. Axonal-anterograde transport of CTB to the ON and SC was visualized directly using fluorescence microscopy. Tissue sections were washed with PBS, mounted with a DAPI-containing mounting medium, and imaged using a Keyence fluorescence microscope (46).

### MB occlusion model.

The MB occlusion model was developed by injecting magnetic beads in the anterior chamber. The magnetic beads block TM outflow, elevating IOP and inducing RGC loss as described previously (21, 72). The experiment was performed in 4-month-old male C57BL/6J mice. Anesthetized animals were given topical eye drops of Tropicamide (0.5%, 1 drop) to dilate the pupil, and a drop of local anesthetic (0.5% proparacaine hydrochloride; SANDOZ) was applied to the cornea. Angled forceps were used to proptose the eye. The cornea was then breached with a glass-pulled micropipette that was guided to the anterior chamber. In total, 1–1.5 μL of 5.8 μm diameter magnetic MBs (Bangs Laboratories) were injected into the anterior chamber of both eyes using a manual micromanipulator (World Precision Instruments [WPI]). A magnet was used to draw the beads in place before withdrawing the micropipette. Injected eyes were then covered in ophthalmic ointment, and mice were placed on a heating pad for recovery. Another group of mice that received PBS served as controls. IOP was measured every week to monitor IOP elevation. We observed stable IOP elevation of more than 4 mm Hg or more in 50%–60% of the eyes injected with MBs; failure to achieve this level of IOP elevation was likely due to failure of the procedure to keep magnetic beads throughout the TM. Accordingly, we selected the mice that developed the expected OHT and utilized them for subsequent PERG analysis, RBPMS staining, and axonal transport analysis.

### Statistics.

Prism 9.0 software (GraphPad) was used for statistical analyses. Data are shown as mean ± SEM. For all experiments, *n* refers to the number of eyes. *P* < 0.05 was considered statistically significant. The student’s *t* test (2-tailed) was used to compare 2 groups. For comparison of different treatments, 2-way ANOVA was used, followed by a Bonferroni post hoc correction. Data analyses were performed in a blinded manner.

### Study approval.

Animal studies were performed in agreement with the guidelines of the Association for Research in Vision and Ophthalmology (ARVO) Statement for the Use of Animals in Ophthalmic and Vision Research. Experimental protocols were approved by appropriate IACUC and Biosafety Committees at the University of California, Irvine.

### Data availability statement.

The datasets used and/or analyzed in the present study are available from the corresponding author upon reasonable request. Supporting data values are included in the Supporting Data Values file.

## Author contributions

GSZ and BRK designed the research studies. BRK, RK, and LL performed key in vivo experiments and analyzed data. JCM performed conscious IOP measurements. YS and LL performed studies related to the MB model. SY, PM, and KP assisted in some experiments. WC and DSK performed RNAScope studies. BRK and GSZ wrote the manuscript. KP assisted in editing it. All authors discussed the results and implications and commented on the manuscript at all stages.

## Supplementary Material

Supplemental data

Unedited blot and gel images

Supporting data values

## Version 1 01/21/2025

In-Press Preview

## Version 2 03/10/2025

Electronic publication
