# Supplementary material for: Impaired axonal transport contributes to neurodegeneration in a Cre-inducible mouse model of myocilin-associated glaucoma
Source: JCI Insight. 2025 Jan 21;10(5):e188710. doi: 10.1172/jci.insight.188710 (PMC11949003; doi:10.1172/jci.insight.188710)
Supplement: Supplemental data [file jciinsight-10-188710-s231.pdf]

Supplemental Information

Kaipa *et al*.

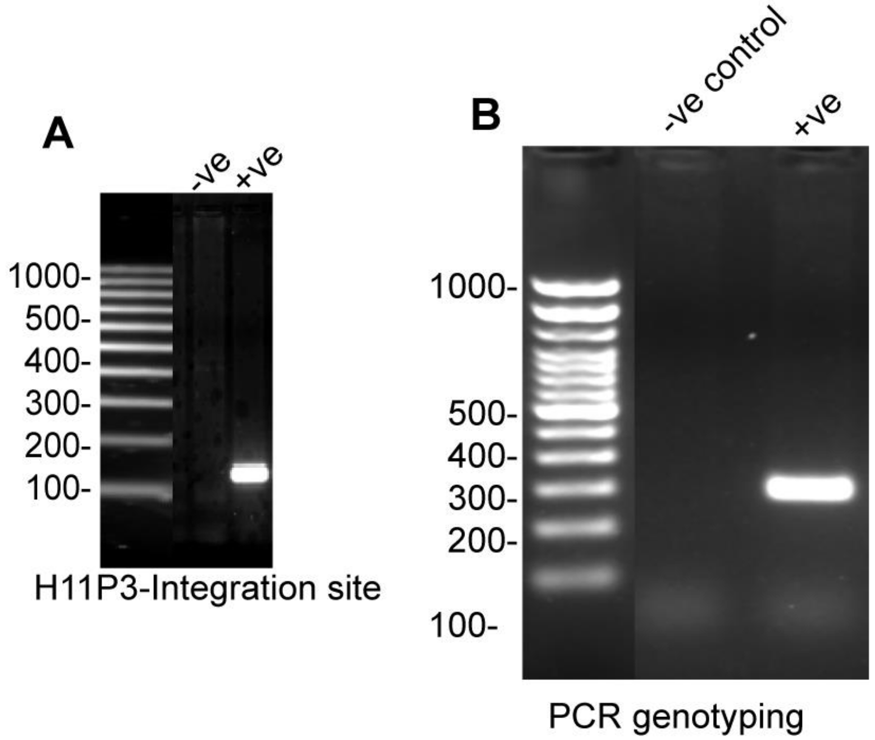

Figure SI-1. Confirmation of transgene integration at the H11 site in *Tg.CreMYOC^{Y437H}* mice. (**A**) PCR was performed using primers specific to the integration site to confirm a site-specific knock-in of the transgene at the H11 site. PCR demonstrated a stable integration of transgene in one founder line. This founder was further bred with C57BL/6J mice, and offspring were utilized for subsequent studies. (**B**) For genotyping, specific primers targeting the human *MYOC* gene and the *DsRed* region were used to detect the presence of the transgene. The positive PCR band at 300 base pairs indicated the presence of the transgene.

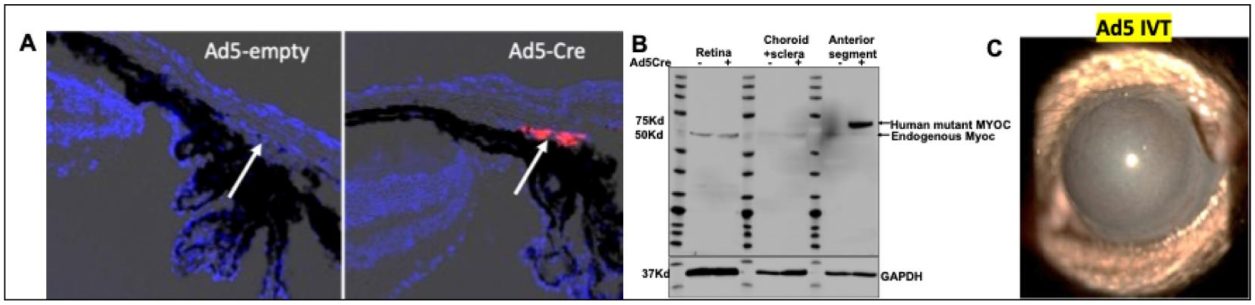

Figure SI-2. Ad5-Cre induces mutant myocilin in the TM cells of *Tg.CreMYOC*^{Y437H} mice. *Tg.CreMYOC*^{Y437H} mice were injected intravitreally with Ad5-empty or Ad5-Cre (2x10*7pfu/eye). (**A**) DsRed (indicating mutant myocilin) was localized to the TM cells of *Tg.CreMYOC*^{Y437H} mice, 5 weeks after injection of Ad5-Cre (n=6 mice), no DsRed expression was found in other ocular tissues. (**B**) Various ocular tissues were harvested from *Tg.CreMYOC*^{Y437H} mice, 5 weeks after injection of Ad5-empty (control) or Ad5-Cre, aliquots of the tissue samples were subjected to Western-blot analysis for myocilin. The Western blots showed the presence of 75-kDa human mutant myocilin exclusively in iridocorneal angle tissues from the Ad5-Cre-injected eyes, whereas endogenous WT myocilin was detected in all the ocular tissues using the myocilin antibody. (**C**) Slit lamp image of mice, 5 weeks after injection with Ad5-Cre, showing that ocular inflammation is associated with Ad5-Cre.

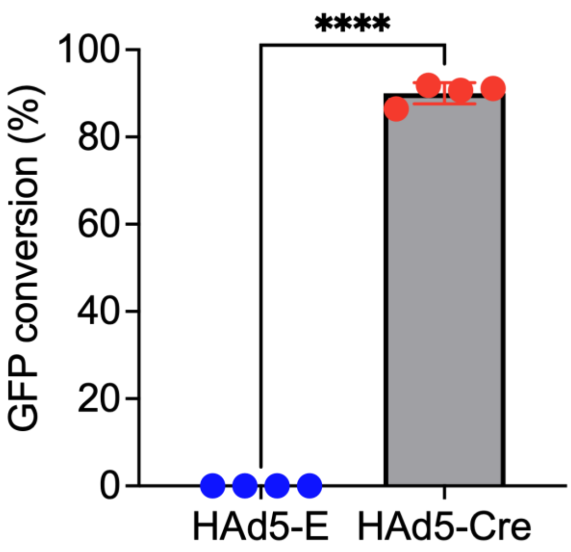

Figure SI-3. HAd5-Cre transduces robust Cre activity in mT/mG fluorescence-based reporter mice. mT/mG reporter mice were injected intravitreally with HAd5-Cre (2 × 10^7 pfu/eye), and the conversion from tdTomato to GFP was examined after one week, using confocal microscopy (n=4). GFP-positive TM cells were counted, and their number was divided by the total number of TM cells present in each section (based on DAPI); the fraction X 100 is represented as % GFP conversion. Over 90% of the TM cells were transduced by HAd5-Cre injection compared to HAd5-E injection ($P < 0.0001$, unpaired two-tailed t-test; mean ± SEM).

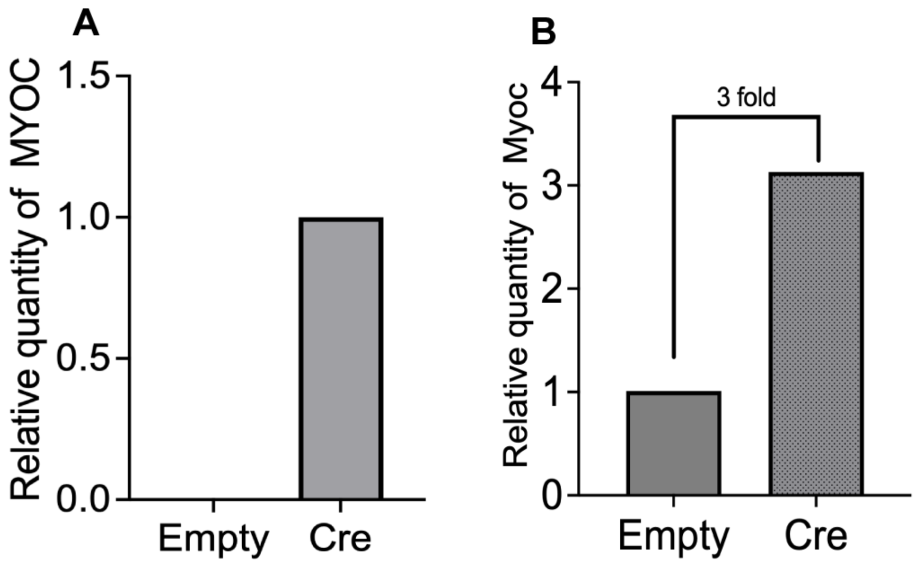

Figure SI-4. An intravitreal injection of HAd5-Cre recombinase induces mutant *MYOC* transcript at a similar level of endogenous *Myoc* in the anterior segment of *Tg.CreMYOC^{Y437H}* mice. HAd5-empty or HAd5-Cre (2x10^7 pfu/eye) were injected intravitreally into *Tg.CreMYOC^{Y437H}* mice and anterior segments from all five mice were pooled together and subjected to qPCR using primers specific to human mutant *MYOC* and endogenous *Myoc*. **A)** Quantitative analysis of *MYOC* mRNA expression levels using qPCR indicated induction of MYOC mRNA exclusively in HAd5-Cre-injected mice (XY graph). **B)** qPCR analysis of endogenous *Myoc* revealed that expression of mutant *MYOC* induced endogenous *Myoc* expression by 3-fold in Cre-injected *Tg.CreMYOC^{Y437H}* mice. In addition, we observed that mutant *MYOC* was expressed at a similar level of endogenous *Myoc* in Cre-injected *Tg.CreMYOC^{Y437H}* mice. *MYOC* transcript was normalized to the housekeeping gene HPRT. N-5 eyes in each group (XY graph).

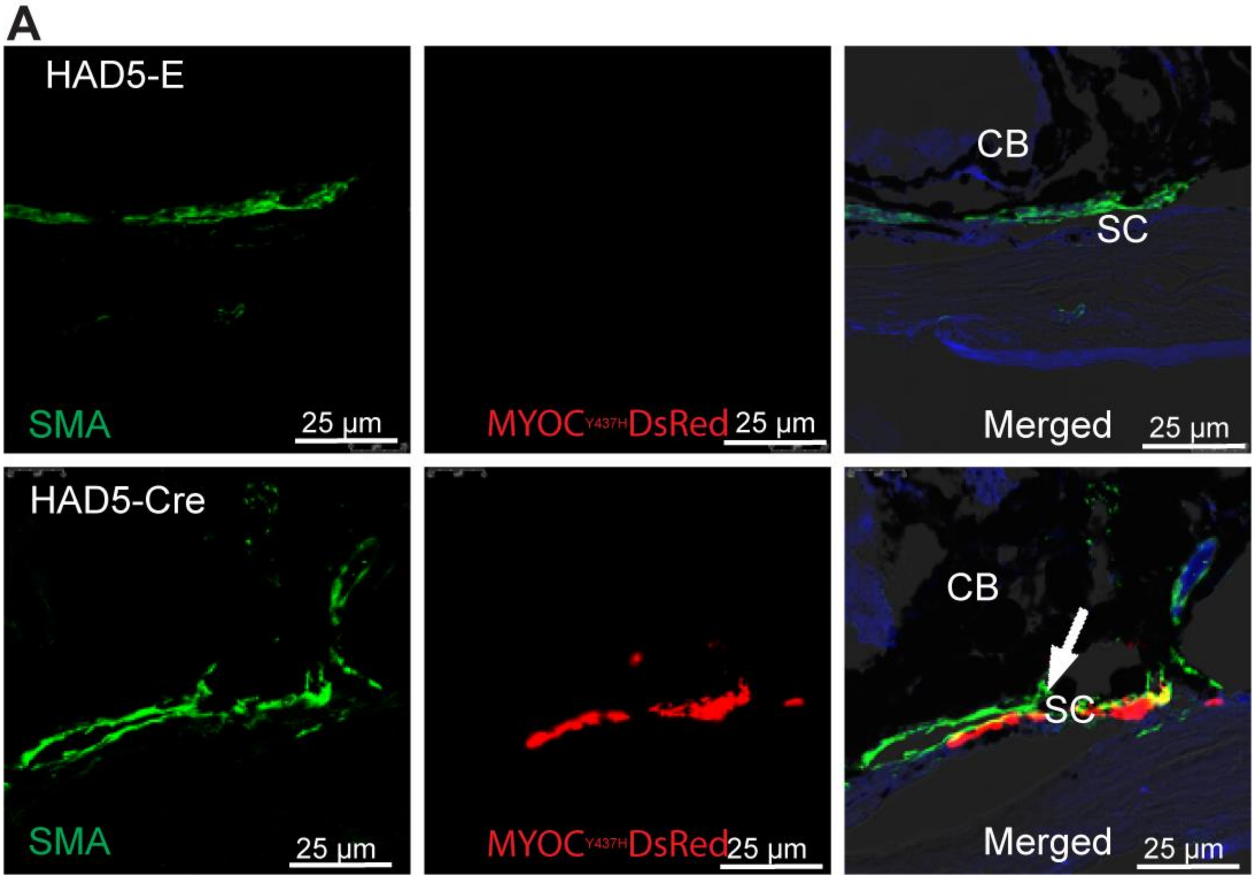

Figure SI-5. Mutant myocilin colocalizes with the TM-specific marker, smooth muscle actin (SMA) in Cre^{+}*Tg.CreMYOC*^{Y437H} mice. Anterior-segment cross sections from Tg.Cre.MYOC^{Y437H} mice, injected with HAd5-empty or HAd5-Cre, were immunostained with antibody against alpha-smooth muscle actin (SMA), which predominantly stains TM and ciliary muscle. We observed strong co-localization of SMA with DsRed-tagged mutant MYOC in the TM region but not in the ciliary muscle region, indicating that mutant myocilin is specifically expressed in the TM (n=3).

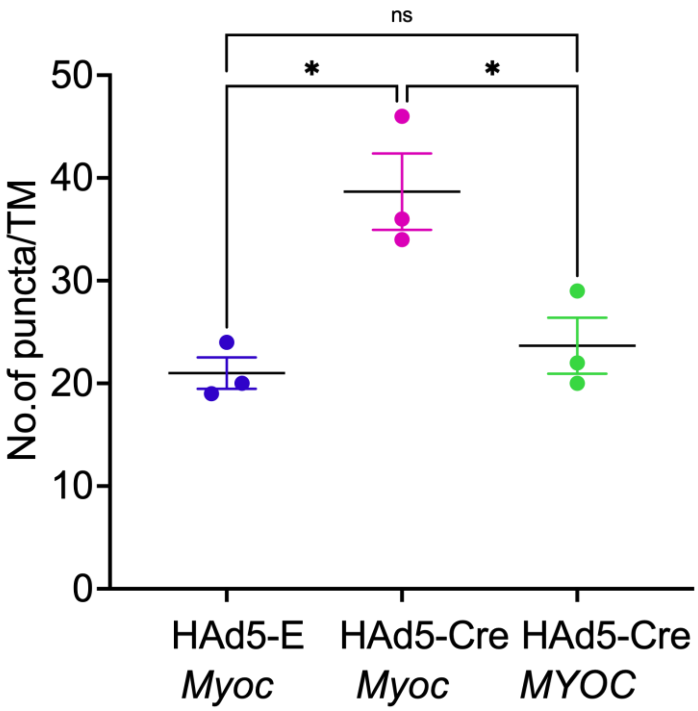

Figure SI-6. Mutant *MYOC* is expressed similarly to endogenous *Myoc* in *Cre⁺Tg.Cre.MYOC*^{Y437H} mice. mRNA transcripts of mutant *MYOC* and endogenous *Myoc* from *Tg.Cre.MYOC*^{Y437H} mice, injected with HAd5-empty or HAd5-Cre, were analyzed via RNA scope, and images were captured using confocal microscopy. The number of fluorescence punta of mutant *MYOC* and endogenous *Myoc* per TM cell was analyzed by manual counting. We observed that mutant *MYOC* is expressed at a similar level to that of endogenous *Myoc*. Expression of mutant MYOC induced endogenous *Myoc* expression in *Tg.Cre.MYOC*^{Y437H} mice injected with HAd5-Cre. N=3 in each group; *P < 0.021; one-way-ANOVA).

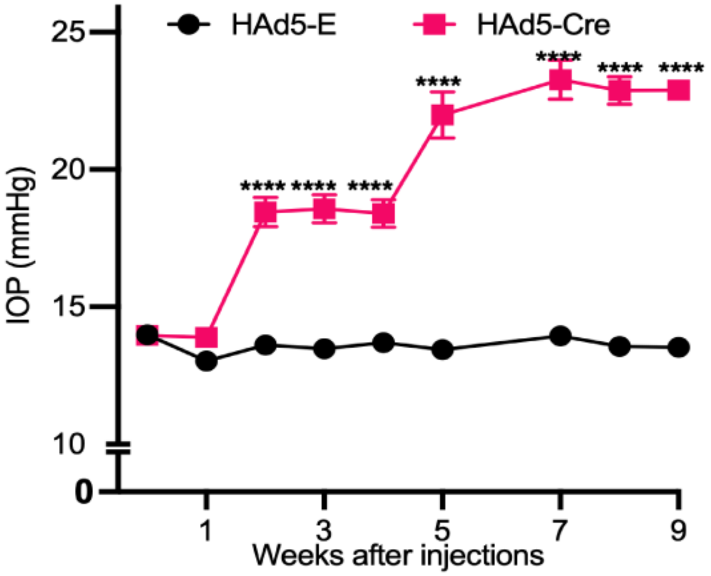

Figure SI-7. Conscious IOP measurements in Cre$^-$ and Cre$^+$ *Tg.CreMYOC^{Y437H}* mice Conscious IOP (without the use of anesthesia) was measured, in a masked manner, in *Tg.CreMYOC^{Y437H}* mice each week from 1 to 9 weeks after intravitreal injection of Had5-empty (control) or Had5-Cre. A significant IOP elevation was observed in HAd5-Cre-injected mice compared to control, starting at 2 weeks after injection. Data are presented as mean ± SEM (n = 8 for HAd5-E; n = 10 for HAd5-Cre), analyzed by 2-WAY ANOVA with multiple comparisons (****$P < 0.0001$).

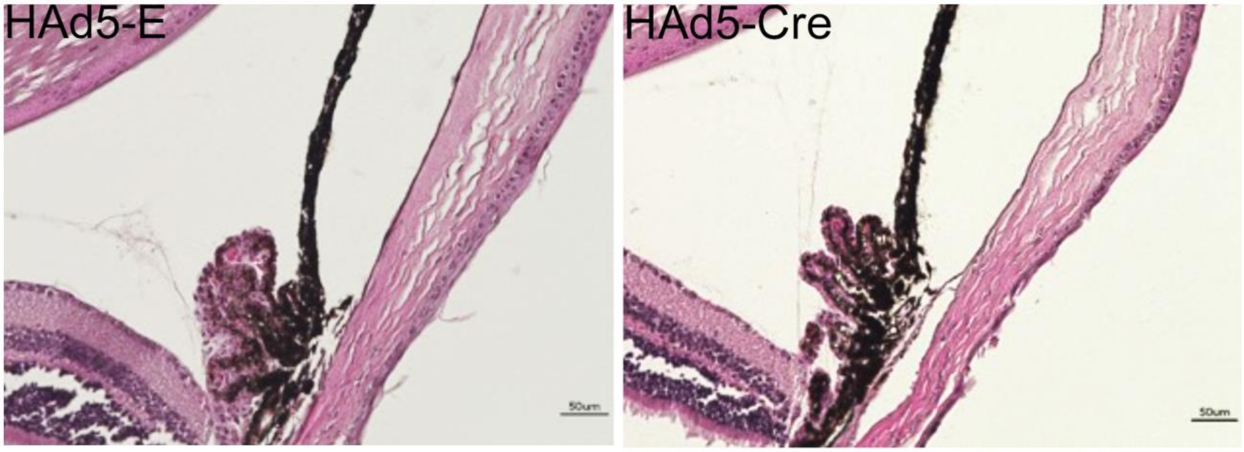

Figure SI-8. Open iridocorneal angle and no gross abnormalities in *Tg.CreMYOC*^{Y437H} mice. *Tg.CreMYOC*^{Y437H} mice were injected with HAd5-empty or Had5-Cre, and *H and E* staining was performed at 5 weeks post-injection. Histological analysis demonstrated open angle and normal ocular structures in both groups of mice (n=3).

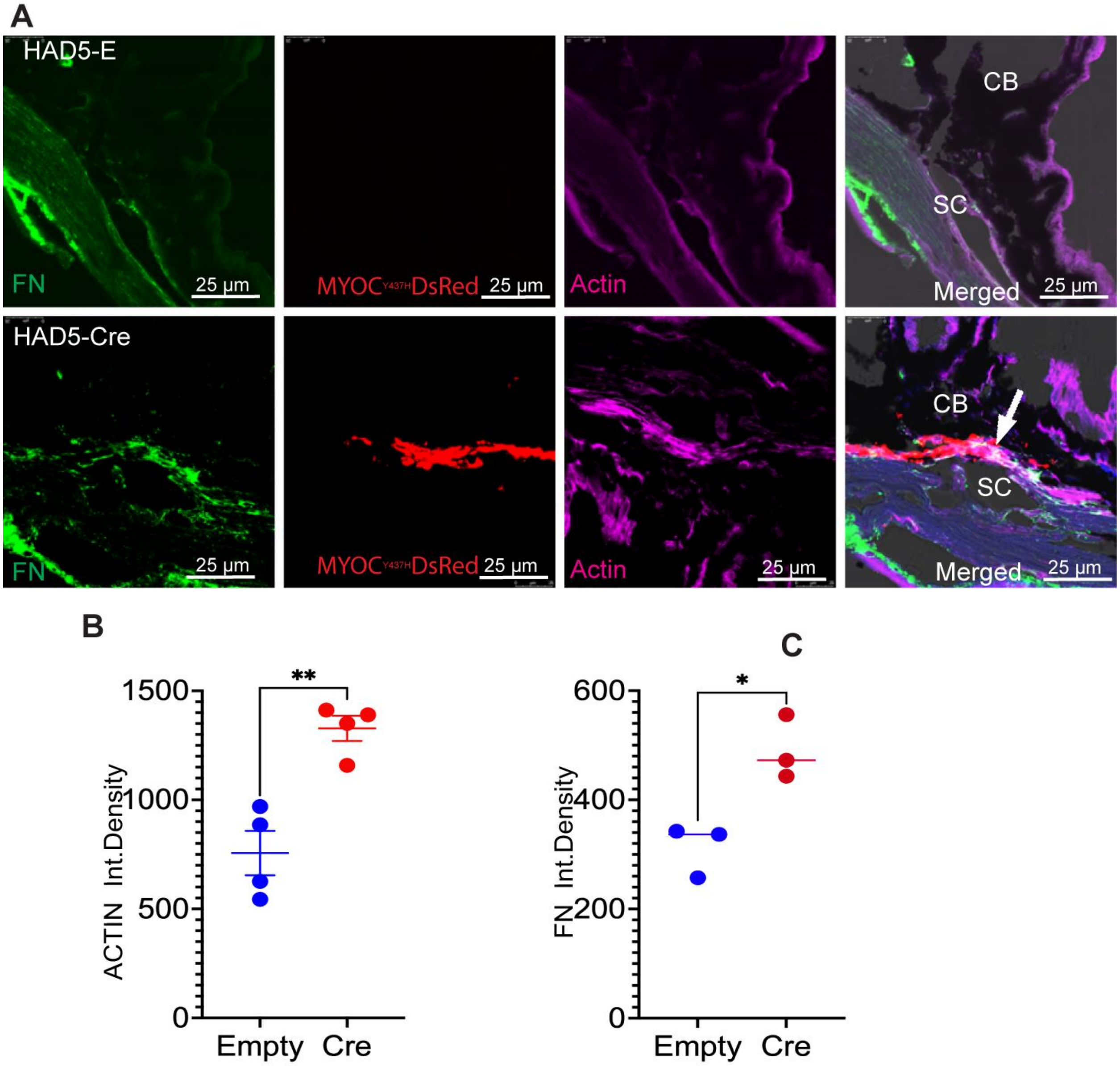

Figure SI-9. Mutant myocilin induces fibronectin and actin in the TM *Cre⁺Tg.CreMYOC*^{Y437H} mice. Increased ECM deposition and actin are associated with TM dysfunction in POAG. We, therefore, examined whether mutant myocilin expression in the TM induces similar changes. Anterior segments from *Tg.CreMYOC*^{Y437H} mice injected with Had5-empty (Cre$^-$, control) or Had5-Cre (Cre$^+$) were immunostained for MYOC (Ds-Red), actin (phalloidin; purple), and fibronectin (green). (**A**) Representative images for control mice (upper panels) and Cre$^+$-mice (lower panels) demonstrated increased fibronectin and actin labeling, which strongly co-localized in the TM (right-most panels). Quantification of integrated intensity for actin (**B**) and fibronectin (**C**)

demonstrated a significant increase in actin (**P = 0.0028, unpaired two-tailed t-test; mean ± SEM, n = 4) and fibronectin (*P = 0.0149, unpaired two-tailed t-test; mean ± SEM, n = 3) levels in *Cre⁺Tg.CreMYOC*^{Y437H} mice compared to the controls.

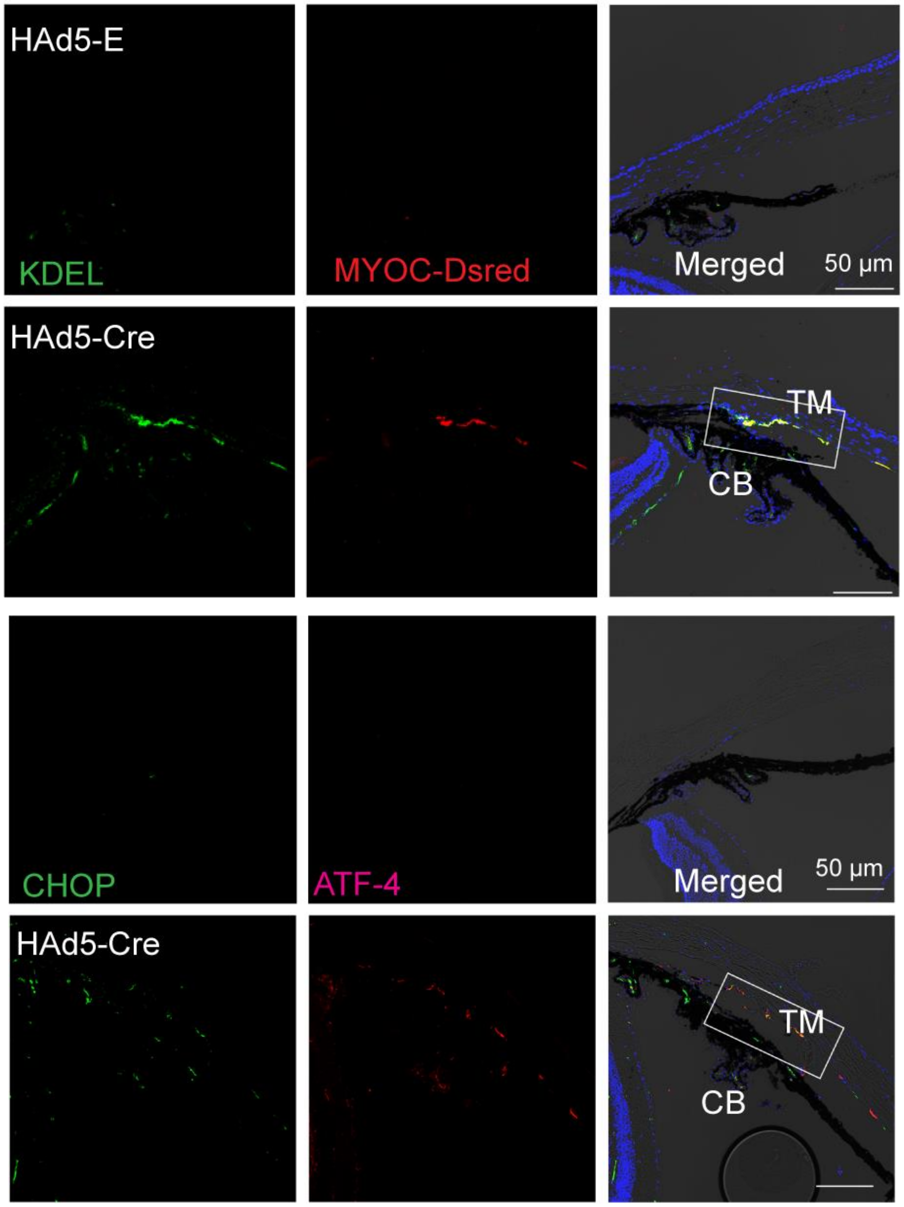

Figure SI-10. Mutant myocilin expression in mouse TM induces ER stress. Anterior segments from *Tg.CreMYOC*^{Y437H} mice, 5 weeks after injection with HAd5-empty or Had5-Cre, were immunostained for various ER stress markers, including KDEL (Top panel) and CHOP+ATF4 (Bottom panel). KDEL (which recognizes GRP78 and GRP94), CHOP, and ATF4 were increased in the TM region of Cre-injected *Tg.CreMYOC*^{Y437H} mice (n=3)

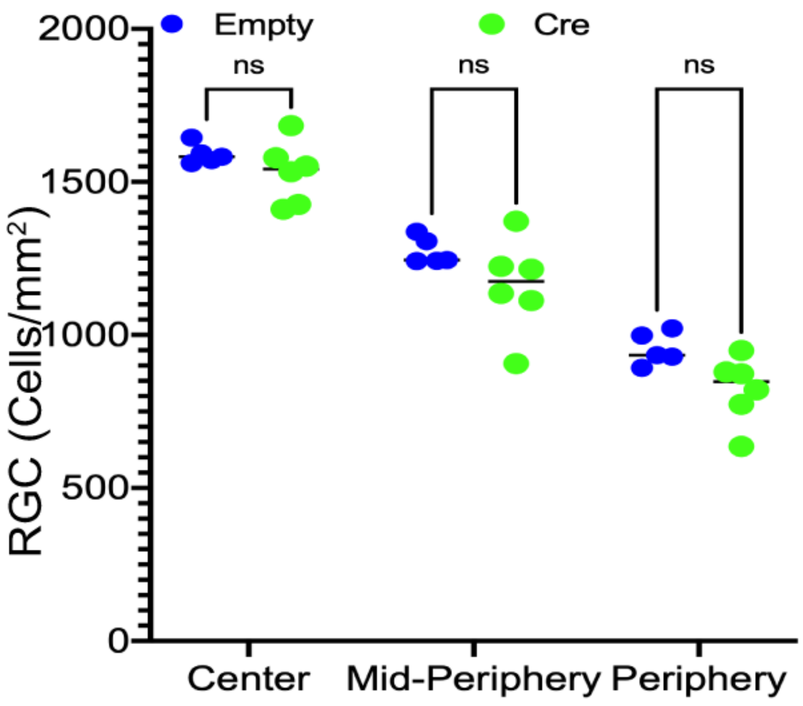

Figure SI-11. RGC analysis in HAd5-Cre-injected *Tg.CreMYOC^{Y437H}* mice, 10 weeks after injection. Whole-mount retina staining with RBPMS was performed in HAd5-Cre or HAd5-empty injected *Tg.CreMYOC^{Y437H}* mice at 10 weeks after injection. The analysis of individual RBPMS-positive RGCs showed no significant RGC loss 10 weeks post-injection. (n=6, 2-WAY ANOVA with multiple comparisons).

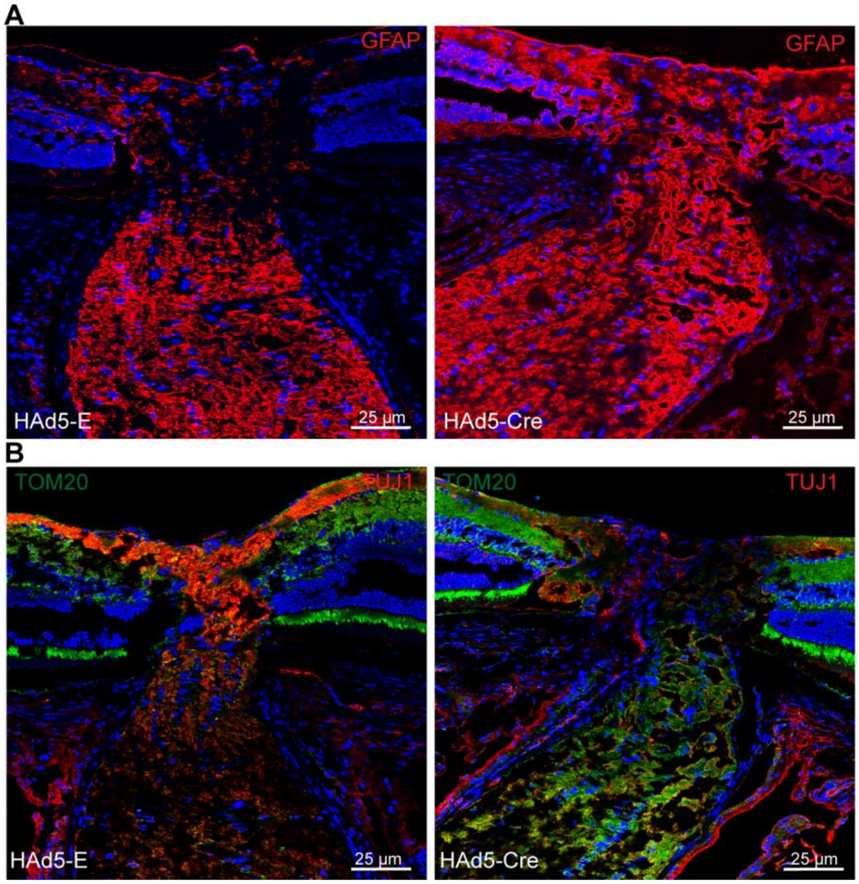

Figure SI-12. IOP elevation leads to increased GFAP and loss of neuronal markers in *Cre*$^{+}$*Tg.Cre.MYOC*^{Y437H} mice. Neurodegenerative changes in ONH of ocular hypertensive *Tg.Cre.MYOC*^{Y437H} mice, after 15 weeks of injection of HAd5-empty or Had5-Cre, were examined for astrocyte activation (GFAP), increased mitochondrial accumulation (TOM 20), and neuronal cytoskeleton changes (Tuj1). **(A**) Immunostaining for GFAP demonstrated gliosis in the ONH region of Cre-injected *Tg.CreMYOC*^{Y437H} mice relative to controls (n=3 for both). (**B**) Immunostaining for TOM20 and Tuj1 (microtubule marker) demonstrated increased mitochondrial accumulation and loss of neuronal cytoskeleton in ON axons of Cre-injected *Tg.CreMYOC*^{Y437H} mice relative to controls (n=3 for both).

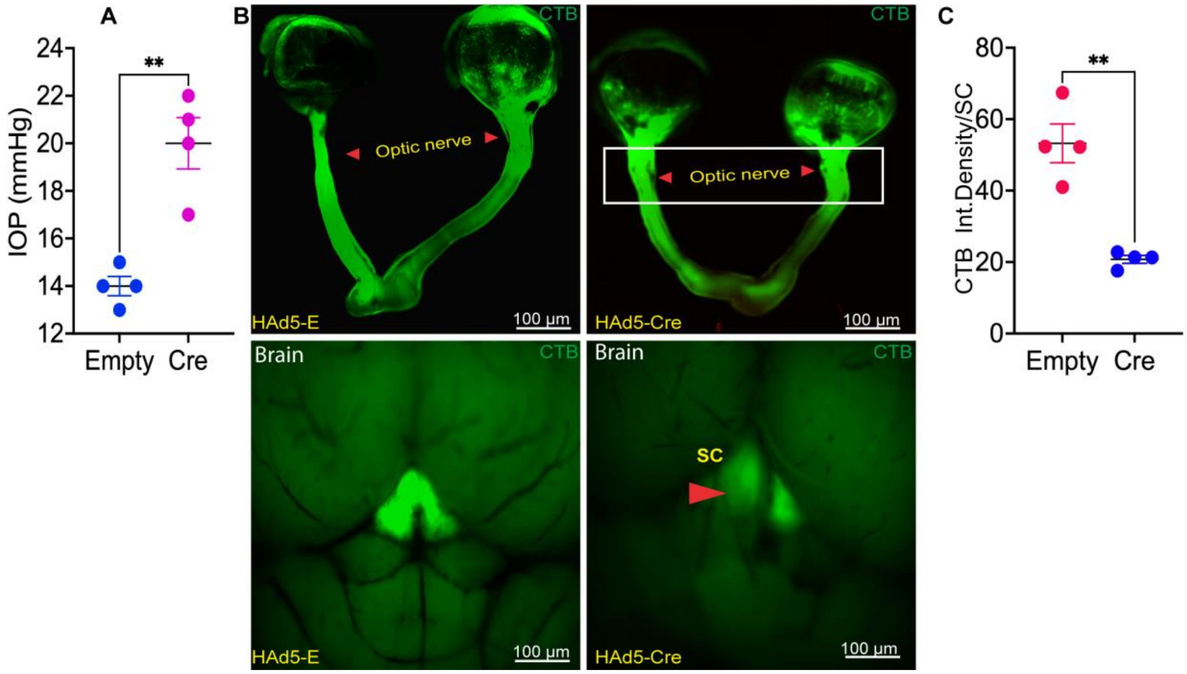

Figure SI-13. Ocular hypertension induces anterograde transport deficits in young *Tg.CreMYOC^{Y437H}* mice. Four-month-old *Tg.CreMYOC^{Y437H}* mice were intravitreally injected with either HAd5-Empty (control) or HAd5-Cre, and anterograde axonal transport mechanisms were assessed. (**A**) IOP measurements revealed significant IOP elevation in the HAd5-Cre mice, 6 weeks post-injection (n = 4; unpaired two-tailed t-test; **P = 0.0020). At 7 weeks post-injection of HAd5-Empty or HAd5-Cre, the mice were then injected intravitreally with cholera toxin B (CTB) to determine the relative transportation of the CTB. (**B**) Representative images of CTB fluorescence are shown for the control mice (left panels) and Cre-injected mice (right panels). In the HAd5-Empty group (left), CTB was transported continuously along the entire length of the optic nerve to the superior colliculus (SC). In contrast, mice injected with Cre (right) displayed a significant transport block at the optic nerve head, and little CTB was detected in the SC. Fluorescence intensity of CTB in the SC was measured and represented graphically in (**C**), showing a 65% loss of CTB transportation to the SC of the Cre-injected mice (n= 4 for HAd5-Empty, n = 3 for HAd5-Cre; unpaired two-tailed t-test; **P=0.0028,). (Note: CTB fluorescence analysis was performed with only those Cre-injected *Tg.CreMYOC^{Y437H}* mice in which the IOP was elevated by 4mmHg or more over the control.)

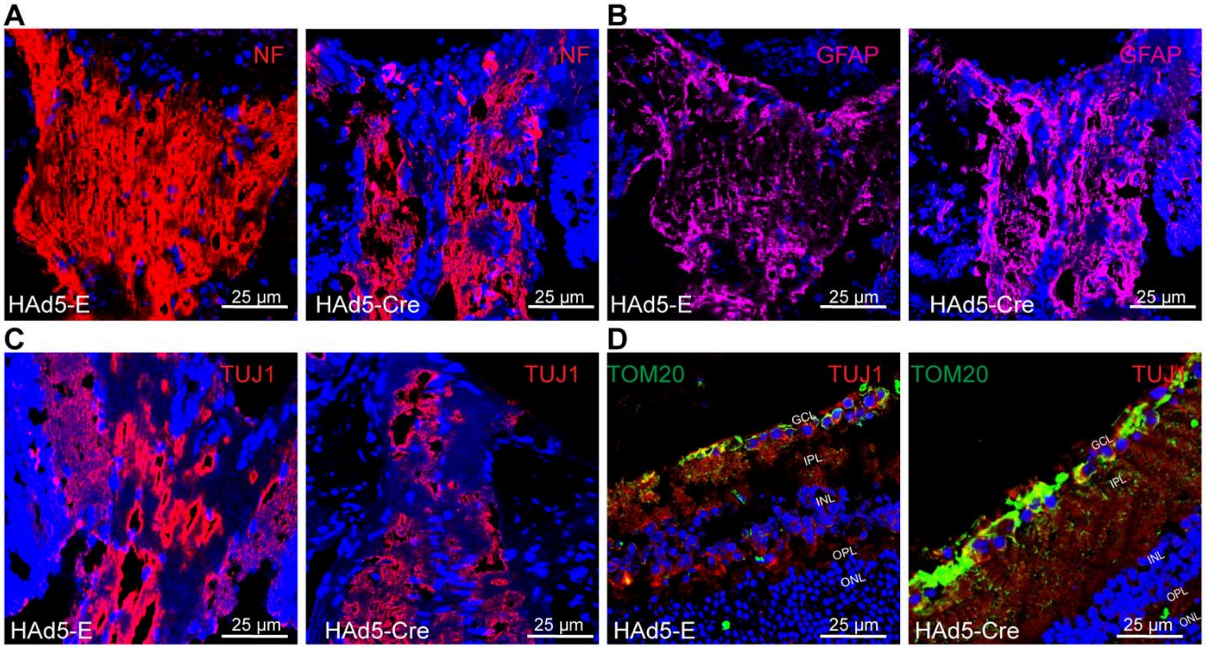

Figure SI-14. Sustained IOP elevation leads to loss of microtubules and neurofilaments in the ONH of *Tg.CreMYOC*^{Y437H} mice. To determine whether impaired axonal transport is associated with loss of cytoskeleton, we utilized *Tg.CreMYOC*^{Y437H} mice at 7 weeks post-injection of HAd5-Cre, which exhibited axonal-transport blockage. Immunostaining of retinal cross-sections revealed reduced neurofilament levels (**A**) and diminished microtubule density (**C**), as indicated by the reduced Tuj1 antibody staining. Increased GFAP levels (**B**) indicated gliosis in the ONH region of the Cre-injected mice. These data indicate that the loss of neurofilament and microtubules is associated with impaired axonal transport in the Cre^{+}*Tg.CreMYOC*^{Y437H} mice. This impairment further results in abnormal accumulation of mitochondria in RGC soma, as evident from increased TOM20 (**D**) at 12 weeks post-injection of HAd5-Cre (n=3).

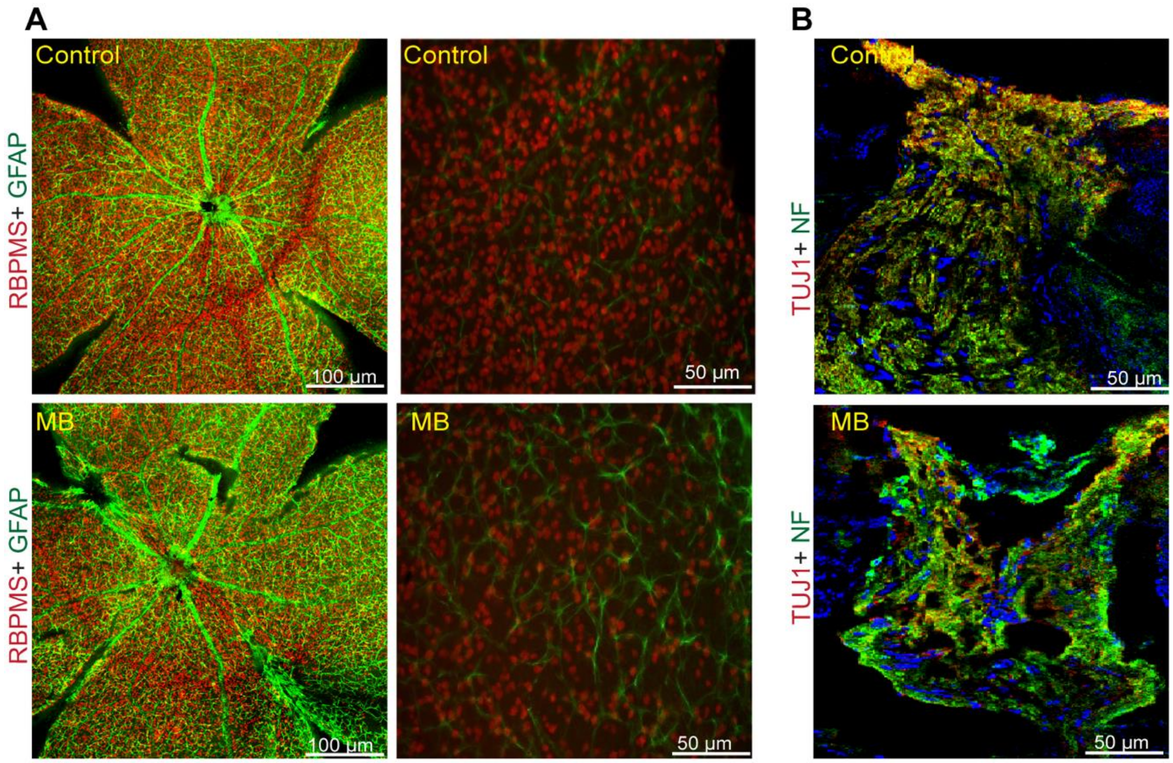

Figure SI-15. Neurodegeneration in a mouse model of microbead-injected mice. C57 mice were injected intracamerally with PBS or microbeads, and neuronal loss was examined in ocular hypertensive mice 6 weeks post-injection. **A)** Representative immunostaining for RBPMS (Red) and GFAP (green) in the central and mid-periphery of the retina from PBS or microbeads-injected mice (Left panel). The right panel shows merged images of RBPMS and GFAP in the periphery of the retina. Although we observed a significant loss of RBPMS-positive RGCs in ocular hypertensive microbead-injected eyes, there was no change in GFAP immunoreactivity in the central and mid periphery of the retina (Right panel). We observed a small increase in GFAP reactivity in the periphery of the microbead-injected retinas in the regions where we observed almost 50% loss of RGCs (n=3 in PBS and 3 in microbead-injected eyes). **B)** Cytoskeleton markers, including Tuj1 and neurofilament, were examined in the ONH region of PBS and microbead-injected mice after 5 weeks of injection. There was no obvious change in Tuj1 or neurofilament staining in the ONH of microbead-injected mice compared to the controls.

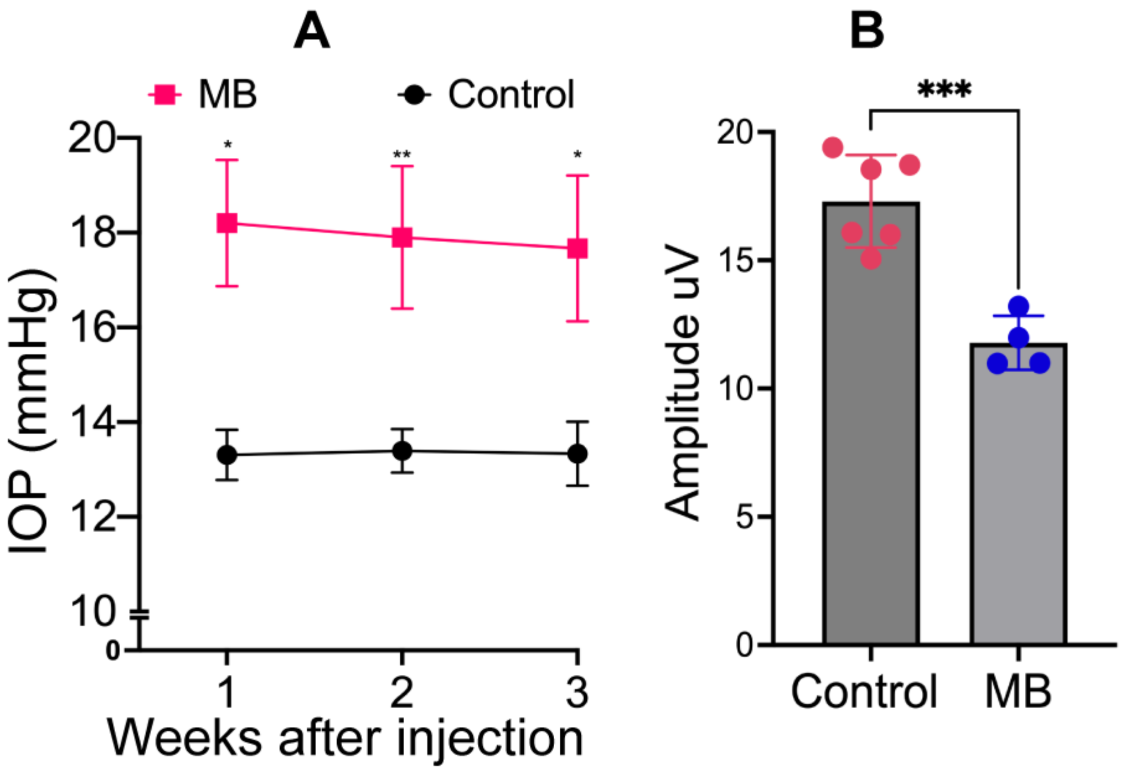

Figure SI-16. Ocular hypertension and functional loss of RGCs in microbead-injected mice. 4-month-old C57 mice were injected with PBS (control) or microbeads (MB) *via* the intracameral route, and IOP was monitored for 3 weeks. (**A**) IOP measurements revealed a significant IOP elevation in the MB-treated mice. Note that IOP is shown only for eyes in which the IOP was elevated by 4mmHg or more over the control (n=6 control, n=4 MB; 2-WAY ANOVA with multiple comparisons). (**B**) PERG analysis was performed on selected ocular hypertensive eyes to determine functional loss of RGCs. PERG analysis demonstrated a significant functional loss of RGCs (n=6 control, n=4 MB; unpaired t-test, two tailed; ***P=0.0006). These mice were further subjected to analysis of CTB transportation. Note that these mice were further utilized for CTB transportation at 4 weeks post-injection.

Methods:

Aqueous humor outflow facility: The outflow facility in Cre^-- and Cre^+-*Tg.CreMYOC*^{Y437H} mice was assessed using the constant-flow infusion method as previously described.[1] Briefly, mice were anesthetized with a ketamine/xylazine solution (100/10 mg/kg), and body temperature was maintained at a physiological level using a 37°C electric heating pad throughout the procedure. Topical ocular proparacaine HCl (0.5%) was administered to induce corneal anesthesia. Anterior chambers were cannulated with 32-gauge ½-inch steel needles (Steriject, Keeler, USA) through the cornea without contacting the iris, anterior lens capsule, or corneal endothelium. The needles were connected to tubing running to a pressure transducer. The opposite end of the transducer was connected by more tubing to a 100-µL microsyringe (Hamilton Co., Reno, NV, USA) loaded into a microdialysis infusion pump, which was filled with sterile PBS. Eyes were infused at a flow rate ranging from 0.1 µL/min to 0.5 µL/min (in 0.1 µL/min increments), and three stabilized pressures at 15-minute intervals were recorded for each flow rate. The aqueous humor outflow facility was then calculated as the reciprocal of the slope (determined by simple linear regression) of the linear part of a plot of mean stabilized pressure (ordinate) *versus* flow rate (abscissa). In a small proportion of cases (13%), data points obtained at flow rates of 0.5 µL/min deviated from linearity. Such points were excluded from the analysis.

Pattern electroretinography (PERG): The RGC function was assessed using a binocular snout-pattern electroretinography (PERG) system (JORVEC Corp., Miami, FL, USA), as described previously.[2] Briefly, mice were anesthetized with an intraperitoneal

injection of ketamine/xylazine mixture (100 and 10 mg/kg, respectively). Anesthetized mice were positioned on a temperature-controllable metal base at a fixed distance (10 cm) from LED monitors and maintained at a constant body temperature (37°C) using a rectal probe. To prevent corneal dryness during recording, a small amount of hypromellose eye drops was applied topically.[2, 3] The PERG was simultaneously derived from each eye using subcutaneous electrodes placed at the snout (active), back of the head (reference), and tail (ground), in response to contrast reversal of gratings generated from two LED screens operating at slightly different frequencies. Two consecutive readings were averaged, and amplitudes (P1–N2) and latencies indicating the RGC-soma function were shown graphically.

Whole-mount retina staining with RBPMS: The total number of RGCs was analyzed using whole-mount retina staining with the RBPMS antibody, as described previously.[2] The enucleated eyes were fixed with 4% PFA for 12 hours at 4 °C. After rinsing the eyes with PBS, the anterior segment was removed, and the retinas were carefully separated from the posterior cup. The isolated retinas were incubated with blocking buffer (PBS containing 10% goat serum and 0.2% Triton X-100) for 12 hours at 4 °C. Subsequently, the retinas were incubated with the RBPMS antibody for 3 days at 4 °C followed by a 2-hour wash in PBS. After washing, the retinas were incubated with a corresponding secondary antibody (goat anti-rabbit 568, 1:500; Invitrogen) for 2 hours at room temperature, washed with PBS three times, and then mounted with a mounting medium containing DAPI nuclear stain. For RGC counting, a minimum of 16 non-overlapping images from the entire retina were captured at 200x magnification using a Keyence

fluorescence microscope (Itasca, IL, USA), and RBPMS-positive cells were counted using ImageJ software.[2-4]

Assessment of optic-nerve degeneration: Degeneration of optic nerve axons was assessed using paraphenylenediamine (PPD) staining, as described previously.[2, 3] In brief, optic nerves were fixed overnight in PBS containing 3% glutaraldehyde/paraformaldehyde at 4°C. The optic nerves were rinsed twice for 10 minutes with 0.1M phosphate buffer and once with 0.1M sodium acetate buffer. Optic nerves were then dehydrated in graded ethanol concentrations. After embedding in resin, transverse semithin sections (1 μm) were cut and stained with 1% PPD for 10 minutes. Ten images without overlap were captured using a confocal Leica microscope and counted manually using Image J software. The sum of surviving axons amounted to ~10% of the total optic nerve cross-sectional area.

Transmission electron microscopy (TEM): To perform TEM imaging of the outflow pathway, 1 μm sections were processed for TEM analysis, as described previously.[2] The animals were perfusion-fixed with 4% PFA, and eyes were enucleated. The anterior chamber and optic nerve were dissected carefully and further fixed with 1.5% paraformaldehyde and 1.5% glutaraldehyde in 100 mM sodium cacodylate buffer (pH 7.2) for at least 24 hours. After aldehyde fixation, the eyes were incubated with 1% osmium tetroxide in 100 mM sodium cacodylate buffer, followed by ethanol dehydration and embedding of the samples in Eponate 812 resin. Sections (1 μm) were cut using an ultramicrotome (EM UC6; Leica) and stained with uranyl acetate and Reynolds lead

citrate. Images were taken with a transmission electron microscope (JEM-1230; JEOL) equipped with a 2K × 2K CCD camera (USC1000; Gatan Inc.), at the University of Iowa Central Microscopy Research Facility.

Visual electrode potential: Mice were dark adapted for eight hours in a light-proof box. The dark-adapted animals were anesthetized with an intraperitoneal injection of a ketamine/xylazine mixture (100, and 10 mg/kg, respectively). Pupils were dilated using a single drop of 0.5% tropicamide for five minutes. Visual evoked potentials (VEPs) were recorded using the Celeris D430 rodent ERG system (Diagnosys LLC, MA, USA) maintained under dark conditions, as described previously.[5] Mice were positioned on a heated platform to regulate body temperature, and corneal moisture was maintained with 1-2% hydroxypropyl methylcellulose. Corneal electrodes integrated with stimulators were applied to lubricated corneas. Ground and reference needle electrodes were subcutaneously inserted into the tail, cheek, and subdermal region of the head, approximating the visual cortex. The midpoint between the ears served as the reference point. Although recording locations were not histologically verified, they were assumed to be consistent based on an impedance of 1.5-10 kΩ. Each eye was subjected to 100 flashes of 1 Hz, 0.05 cd s/m² white light delivered through corneal stimulators. Recordings were captured for 300 ms at a sampling rate of 2000 Hz. Two trials consisting of 50 sweeps were performed for each mouse. VEP data were filtered with low and high-frequency cutoffs of 1.25 Hz and 100 Hz, respectively. The amplitude of N1 was calculated as the distance from the baseline to the most pronounced negative peak, while P1 amplitude was determined from the deepest negative peak to the subsequent positive

peak. Latency was defined as the interval between stimulus onset and the initial response peak.[6]

References:

1. Millar, J.C., A.F. Clark, and I.H. Pang, *Assessment of aqueous humor dynamics in the mouse by a novel method of constant-flow infusion.* Invest Ophthalmol Vis Sci, 2011. **52**(2): p. 685-94.
2. Maddineni, P., et al., *CNS axonal degeneration and transport deficits at the optic nerve head precede structural and functional loss of retinal ganglion cells in a mouse model of glaucoma.* Mol Neurodegener, 2020. **15**(1): p. 48.
3. Kasetti, R.B., et al., *ATF4 leads to glaucoma by promoting protein synthesis and ER client protein load.* Nat Commun, 2020. **11**(1): p. 5594.
4. Kasetti, R.B., et al., *Autophagy stimulation reduces ocular hypertension in a murine glaucoma model via autophagic degradation of mutant myocilin.* JCI Insight, 2021. **6**(5).
5. Du, S.W., et al., *Conditional deletion of miR-204 and miR-211 in murine retinal pigment epithelium results in retinal degeneration.* J Biol Chem, 2024. **300**(6): p. 107344.
6. Liu, S., et al., *An optimized procedure to record visual evoked potential in mice.* Exp Eye Res, 2022. **218**: p. 109011.
