# Supplementary material for: Impaired axonal transport contributes to neurodegeneration in a Cre-inducible mouse model of myocilin-associated glaucoma
Source: JCI Insight. 2025 Jan 21;10(5):e188710. doi: 10.1172/jci.insight.188710 (PMC11949003; doi:10.1172/jci.insight.188710)
Supplement: Unedited blot and gel images [file jciinsight-10-188710-s232.pdf]

Raw Western blot and PCR gel images

Kaipa et al

Uncut and unprocessed images of Western blot used in Figure 1E and 3C

Uncut and unprocessed images of Western blot used in Figure 1 E

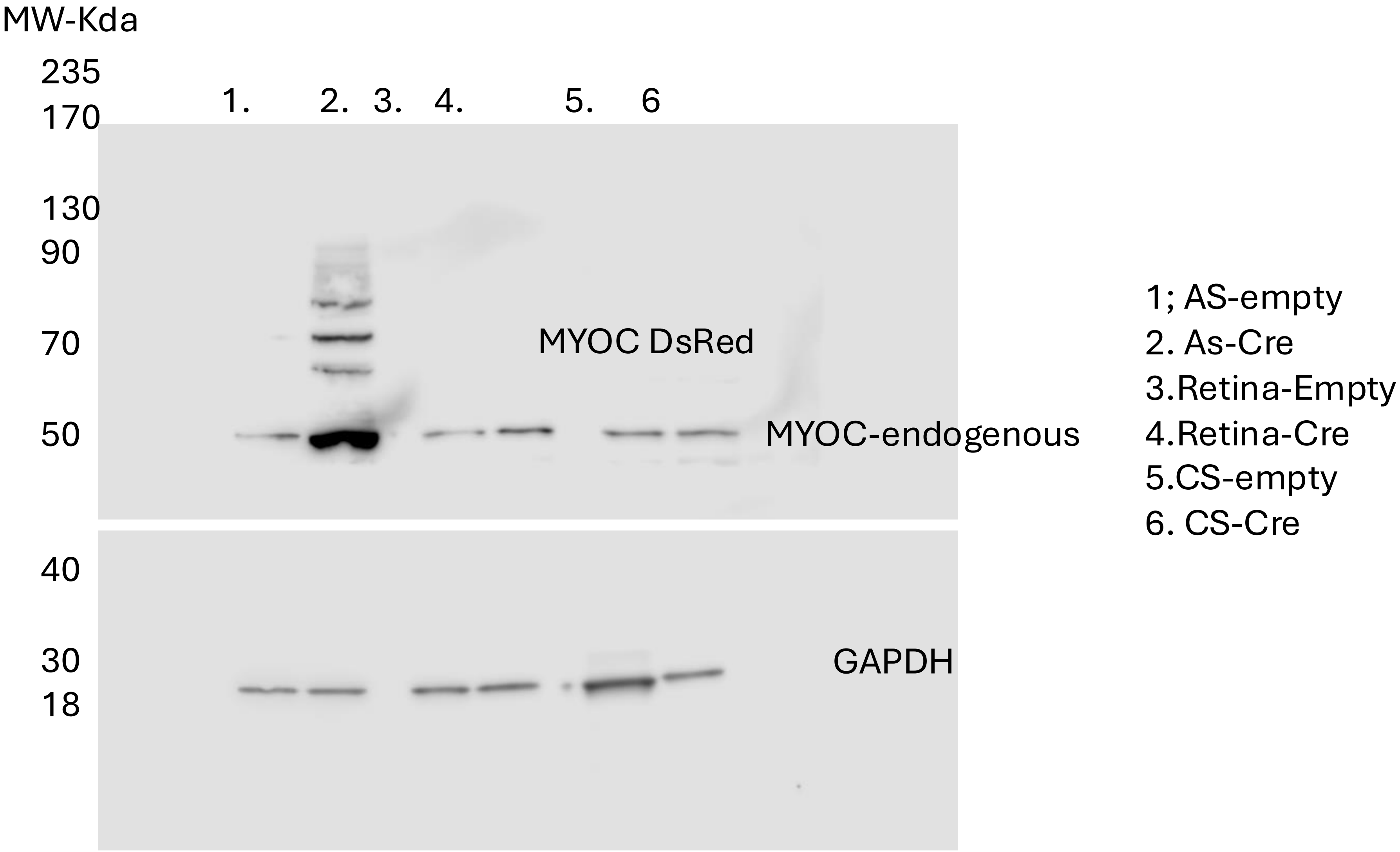
MW-Kda
235
170
130
90
70
50
40
30
18
1.
2.
3.
4.
5.
6
MYOC DsRed
MYOC-endogenous
GAPDH
1; AS-empty
2. As-Cre
3.Retina-Empty
4.Retina-Cre
5.CS-empty
6. CS-Cre

Uncut and unprocessed images of Western blot used in Figure 3 C (n=3)

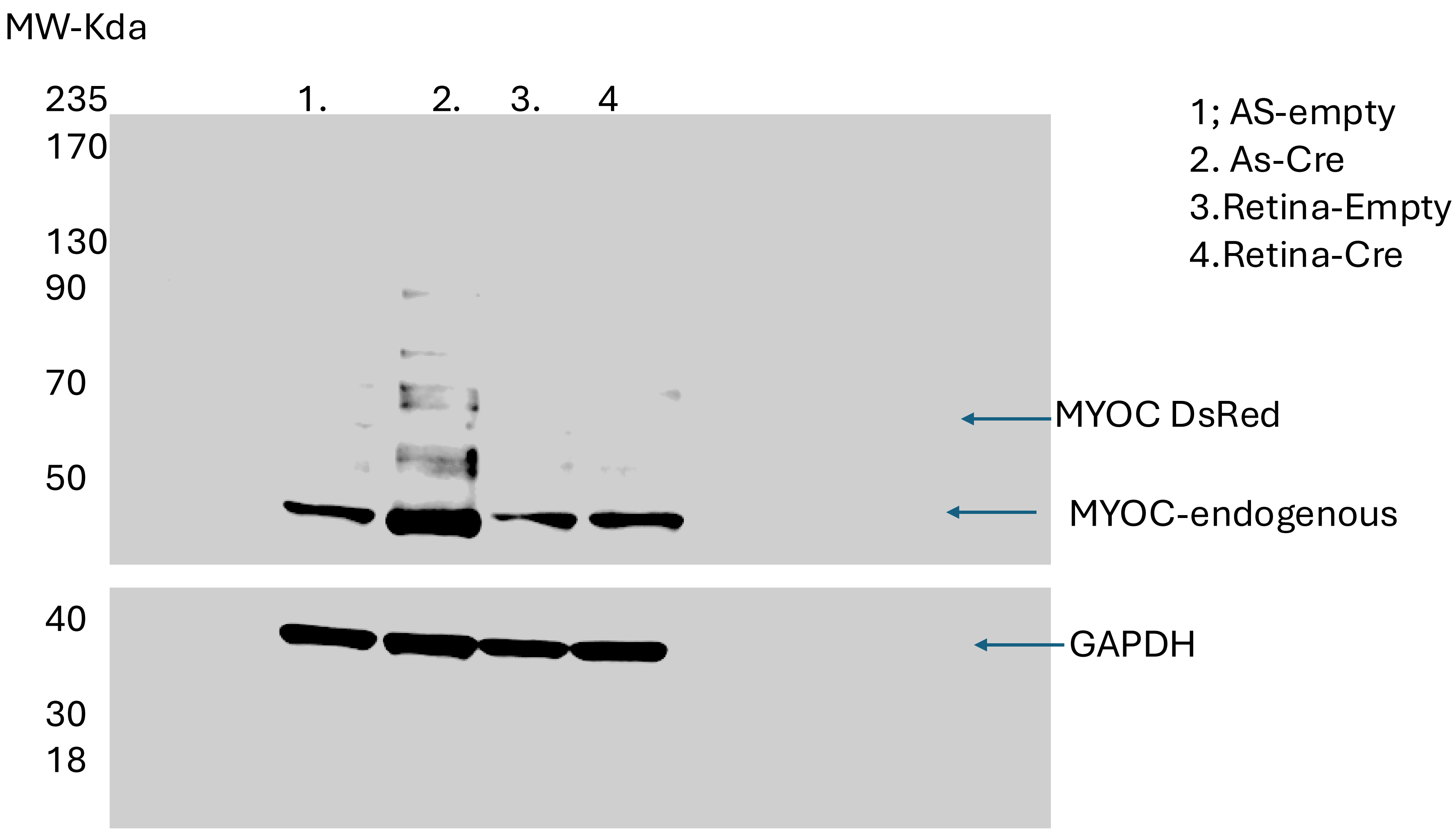
MW-Kda
235
170
130
90
70
50
40
30
18
1.
2.
3.
4
1; AS-empty
2. As-Cre
3.Retina-Empty
4.Retina-Cre
MYOC DsRed
MYOC-endogenous
GAPDH

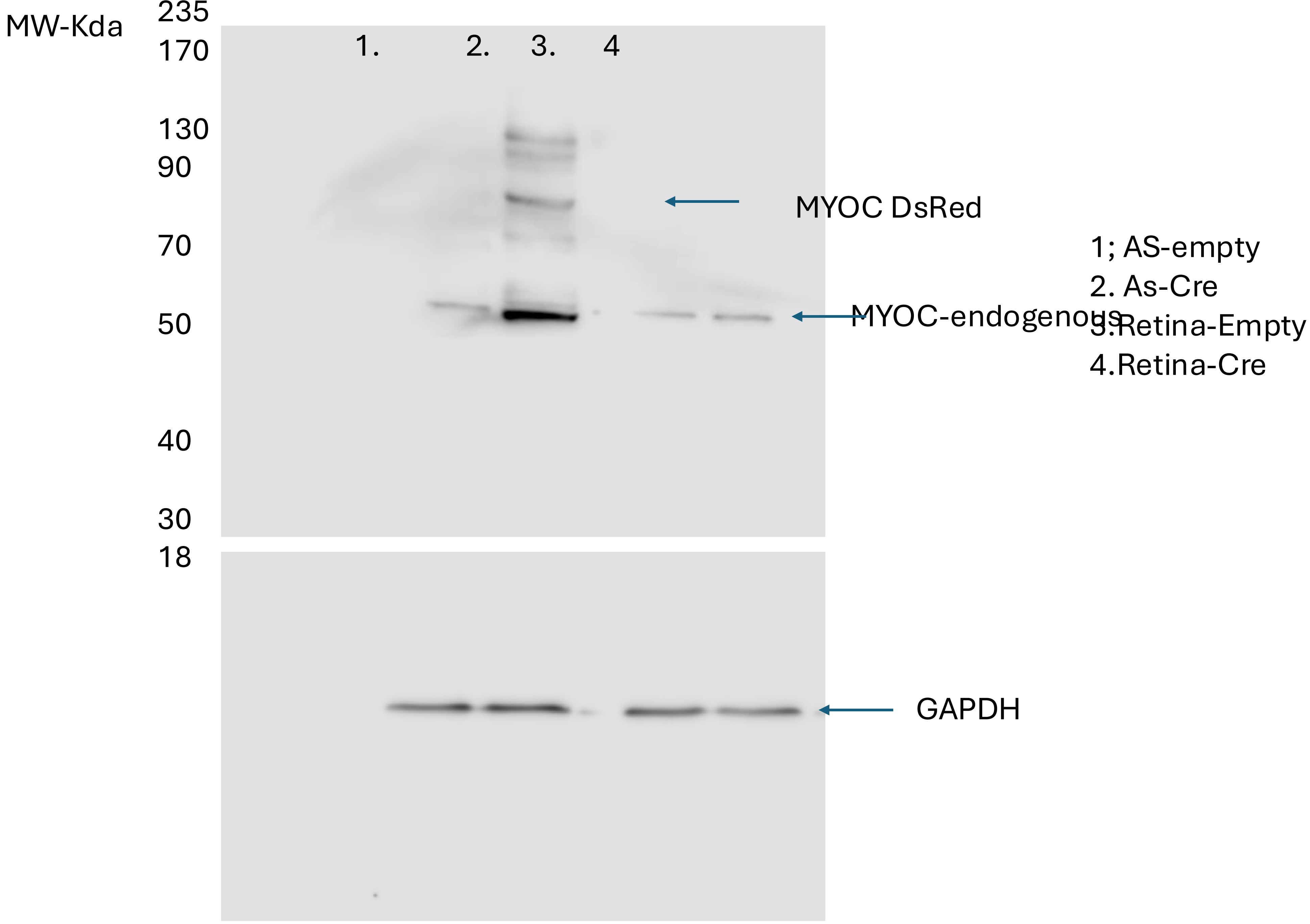
MW-Kda
235
170
130
90
70
50
40
30
18
1.
2.
3.
4
MYOC DsRed
MYOC-endogenous
GAPDH
1; AS-empty
2. As-Cre
3.Retina-Empty
4.Retina-Cre

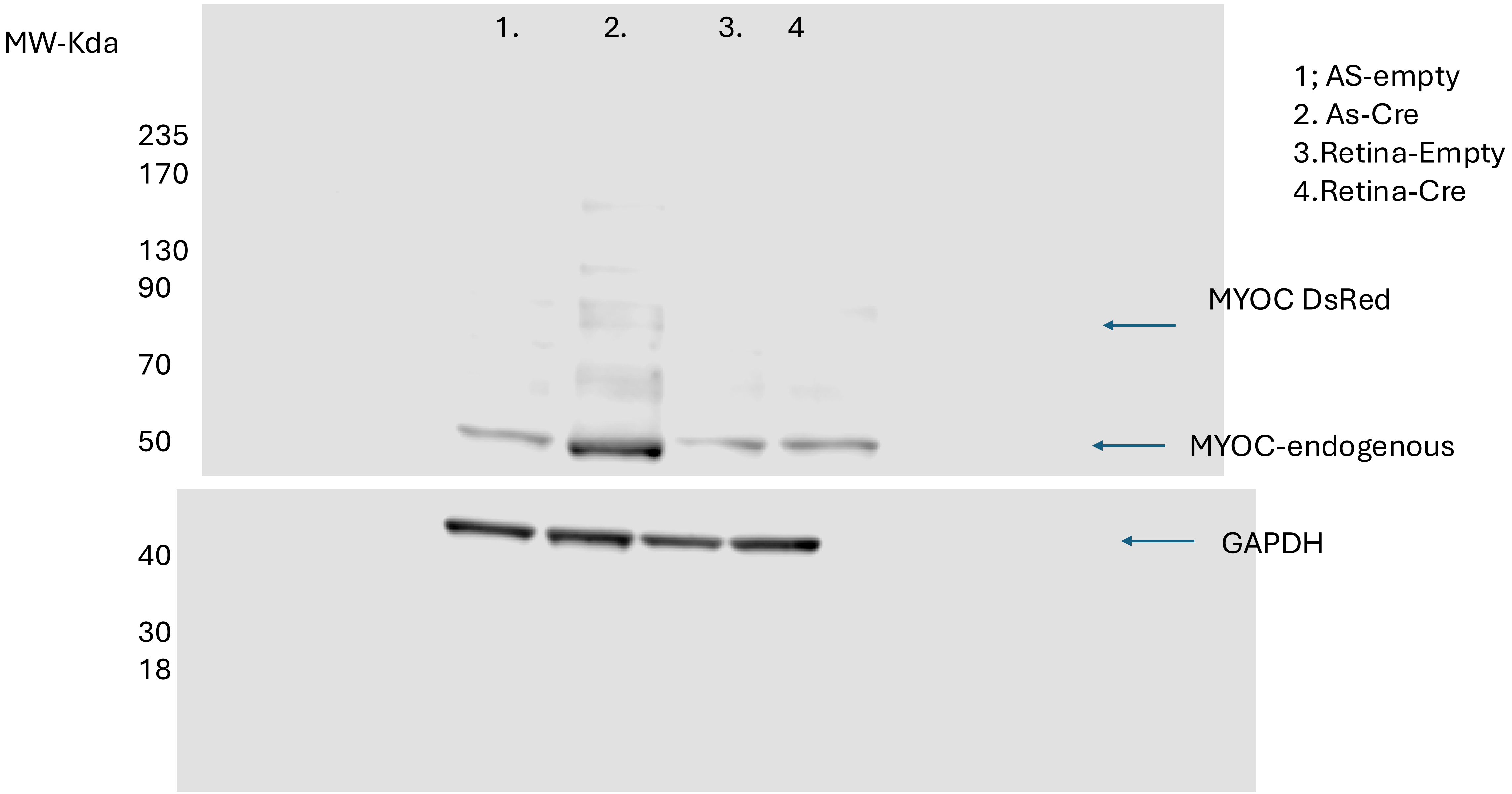
MW-Kda
1.
2.
3.
4
235
170
130
90
70
50
40
30
18
1; AS-empty
2. As-Cre
3.Retina-Empty
4.Retina-Cre
MYOC DsRed
MYOC-endogenous
GAPDH

Uncut and unprocessed images of Western blot used in Figure 3 C (n=3)

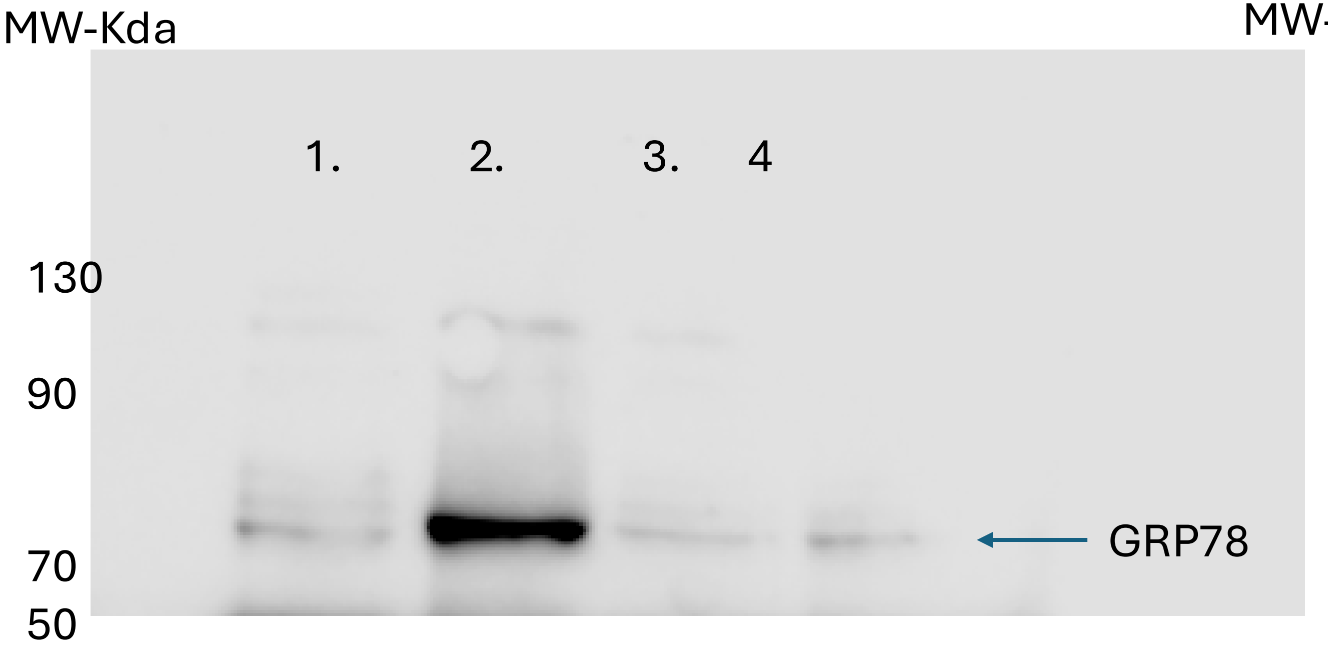

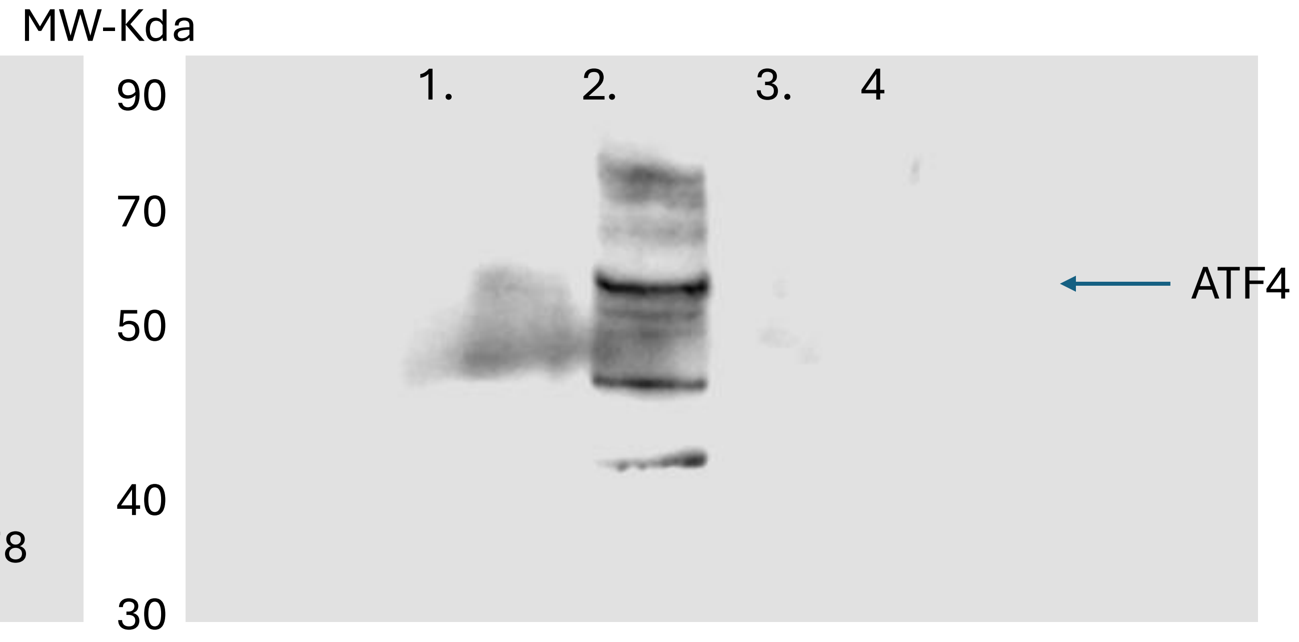

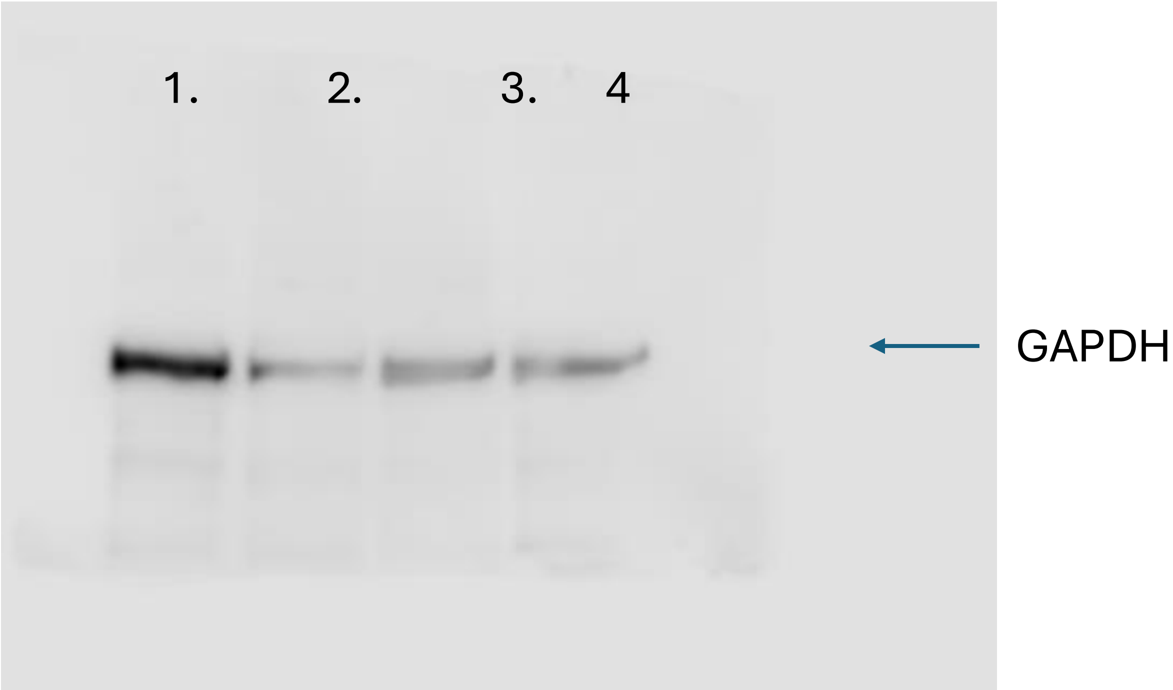

1; AS-empty
2. As-Cre
3.Retina-Empty
4.Retina-Cre

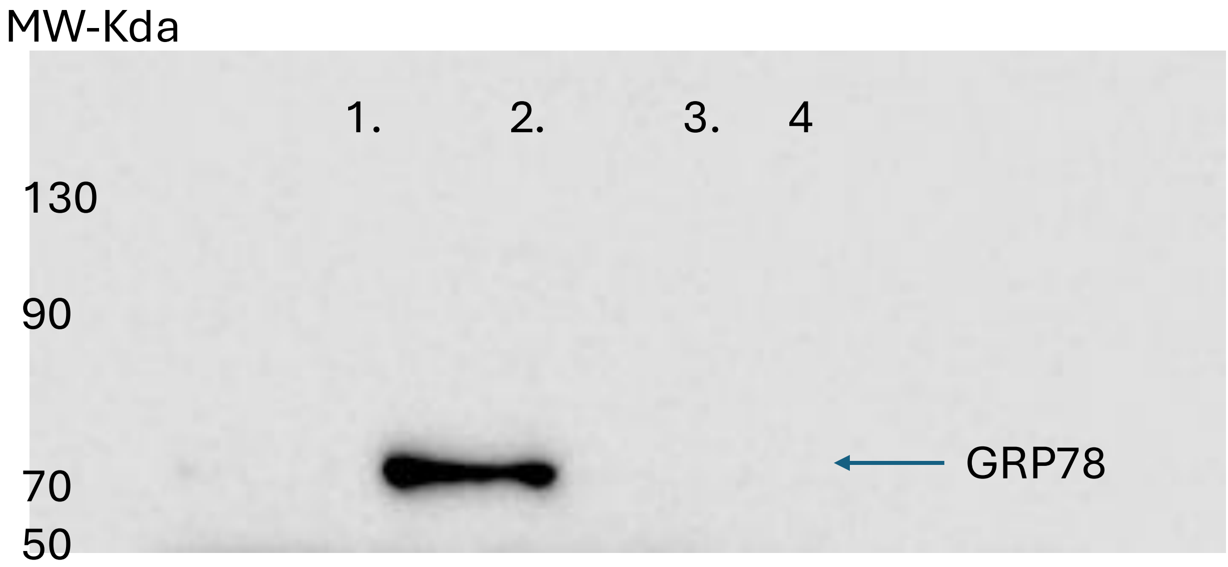

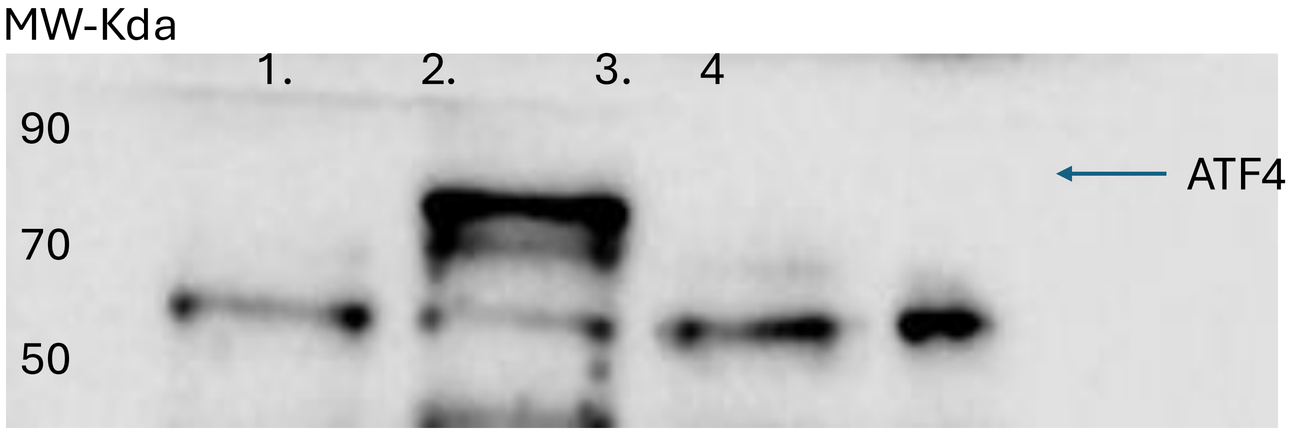

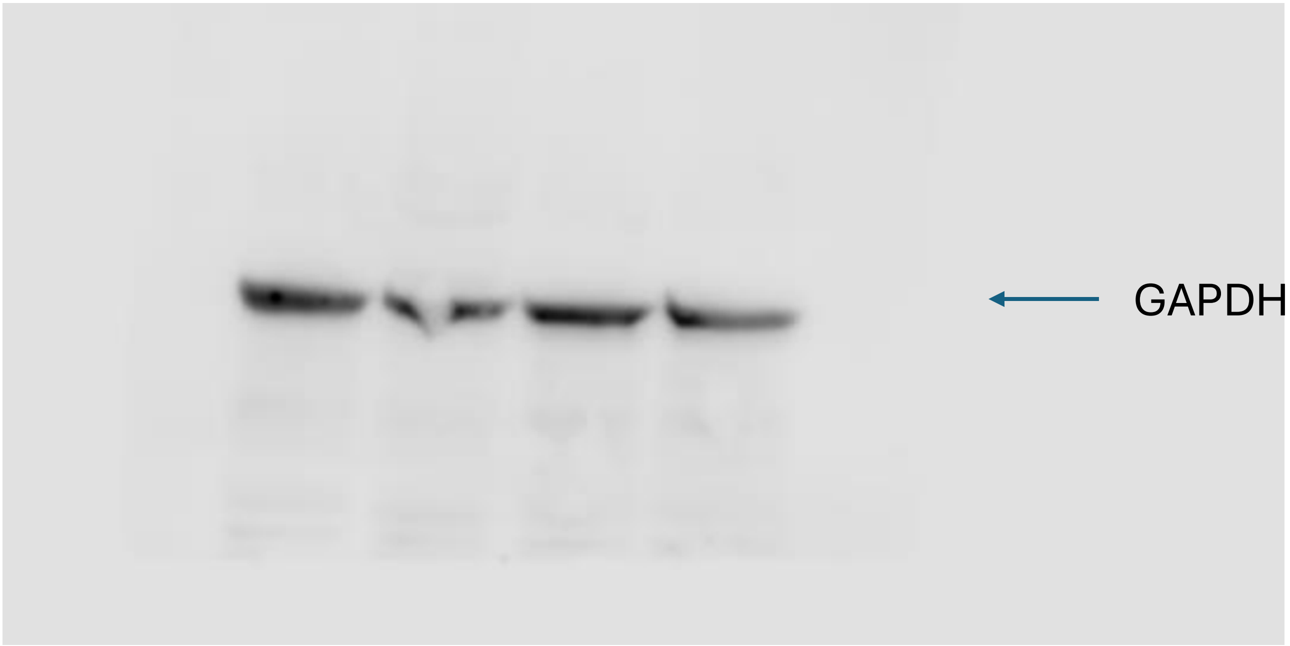

1; AS-empty
2. As-Cre
3.Retina-Empty
4.Retina-Cre

MW-Kda

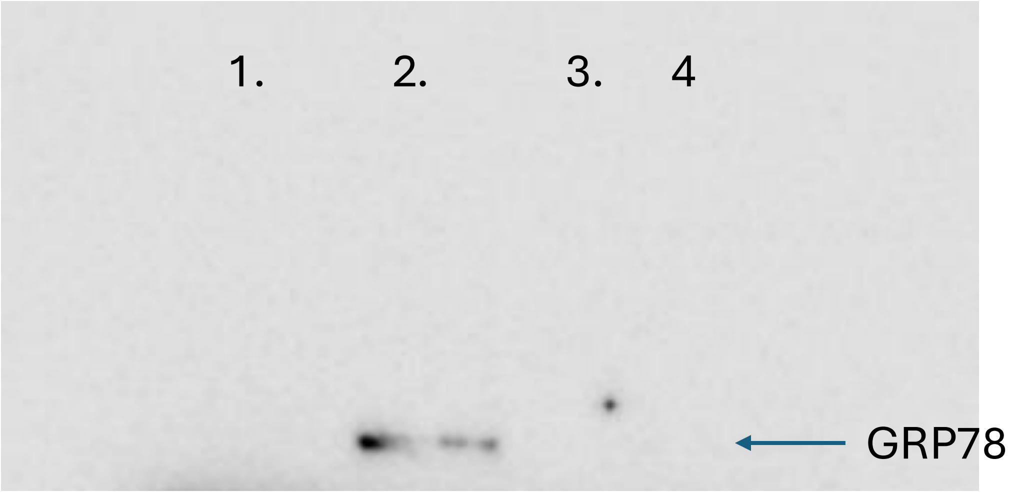

GRP78

MW-Kda

1. 2. 3. 4

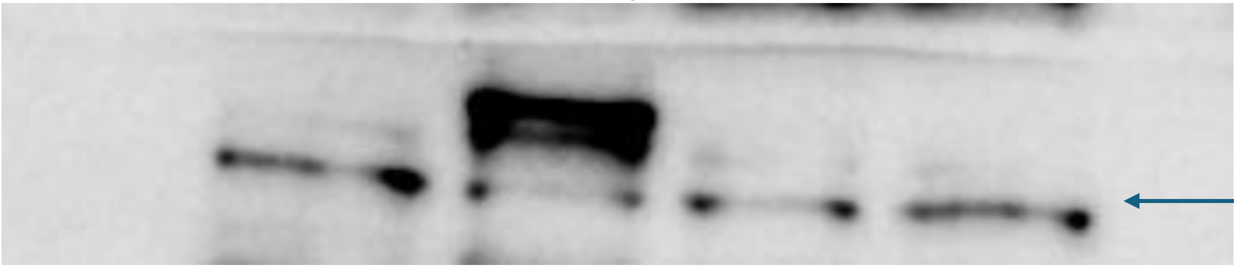

ATF4

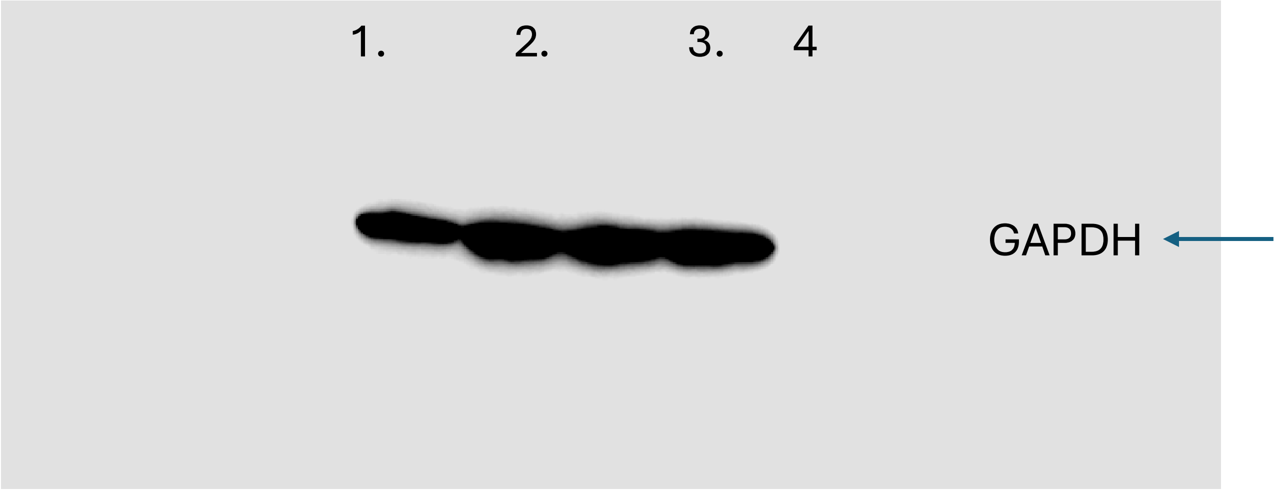

1; AS-empty
2. As-Cre
3.Retina-Empty
4.Retina-Cre

Uncut and unprocessed images of PCR gel for supplementary information Fig 1B

PCR genotyping

1 2. 3. 4. 5. 6

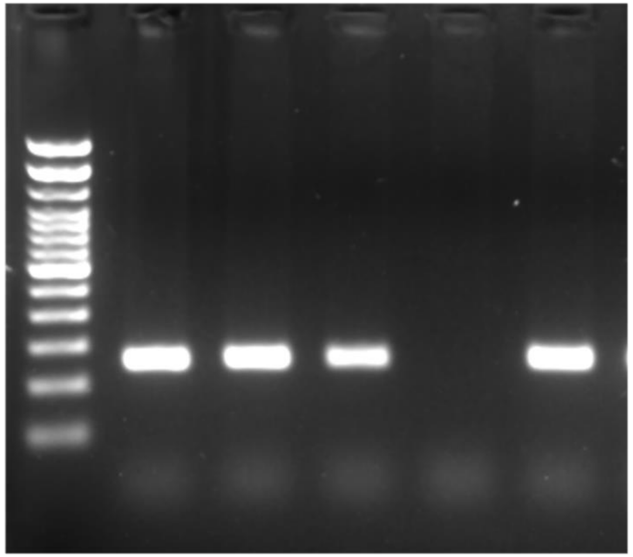

1.+Ve
2. +ve
3.+ve
4.+ve
5. _ve
6. +6

Used 5 and 6 a

Uncut and unprocessed images of PCR gel for supplementary information Fig 1A

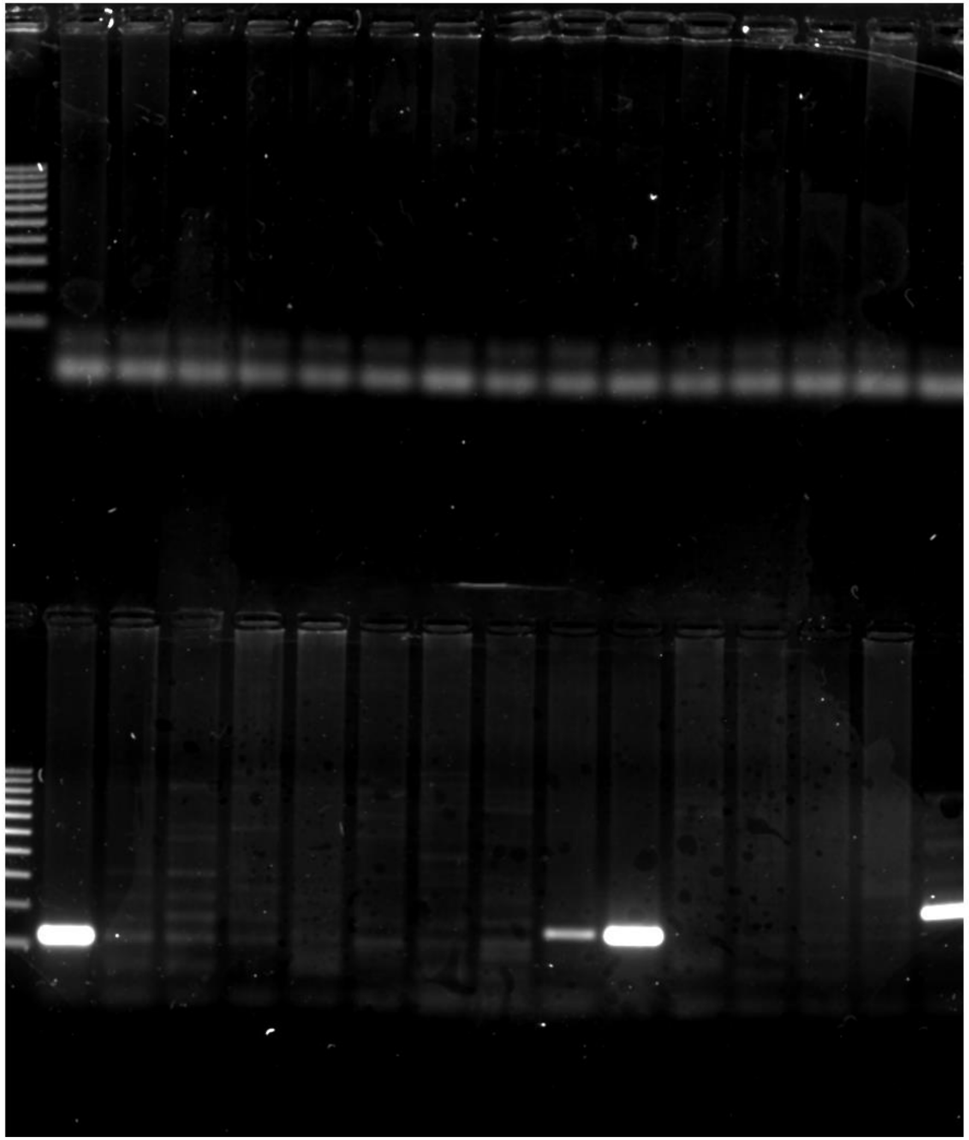

Used only positive and negative for *transgene at the H11 site conformation SI Fig 1A: used last 2 lanes.*
